# Genome-Wide Identification, Characterization of the *ORA* (Olfactory Receptor Class A) Gene Family, and Potential Roles in Bile Acid and Pheromone Recognition in Mandarin Fish (*Siniperca chuatsi*)

**DOI:** 10.3390/cells14030189

**Published:** 2025-01-26

**Authors:** Xiaoru Dong, Maolin Lv, Ming Zeng, Xiaochuan Chen, Jiale Wang, Xu-Fang Liang

**Affiliations:** 1 College of Fisheries, Chinese Perch Research Center, Huazhong Agricultural University, Wuhan 430070, China; xiaorudong@webmail.hzau.edu.cn (X.D.); zengming_0601@outlook.com (M.Z.); cxc2022308@163.com (X.C.); jialewang@webmail.hzau.edu.cn (J.W.); 2 Engineering Research Center of Green Development for Conventional Aquatic Biological Industry in the Yangtze River Economic Belt, Ministry of Education, Wuhan 430070, China; 3 Hubei Hongshan Laboratory, College of Fisheries, Huazhong Agricultural University, Wuhan 430070, China; lvmaolin@webmail.hzau.edu.cn

**Keywords:** *Siniperca chuatsi*, *ORA* gene family, gene structure, expression patterns, cellular localization, molecular docking, pheromones

## Abstract

The *ORA* (olfactory receptor class A) gene family in teleosts is related to the *V1R* (vomeronasal 1 receptors) family in mammals and plays a key role in odor detection. Although *ORA* genes have been identified in several teleosts, their characteristics in mandarin fish (*Siniperca chuatsi*) have not been explored. In this study, we conducted a comprehensive genomic analysis of the mandarin fish and discovered a complete *ORA* gene family consisting of five members located on chromosome 2 (*ORA1*, *ORA2*, *ORA3*, *ORA4*) and chromosome 16 (*ORA6*). Phylogenetic, synteny, and gene structure analyses revealed typical exon–intron conservation with strong evidence of purifying selection. Tissue expression analysis showed distinct expression profiles for each *ORA* gene, with some showing sexual dimorphism in specific tissues. The expression of *ORA1* and *ORA2* in the olfactory epithelium exhibits sexual dimorphism, while *ORA3* shows sexual dimorphism in the brain. *In situ* hybridization confirmed that *ORA1*, *ORA2*, *ORA3*, and *ORA6* are expressed in the microvillar sensory neurons of the olfactory epithelium, while *ORA4* is expressed in crypt cells. Additionally, molecular docking simulations indicated that the five ORA proteins have a high binding affinity with seven bile acids (LAC, GLAC, CA, TLCA, 3-KLCA, 7-KLCA, and 12-KLCA), with ORAs showing stronger binding affinity with LCA and CA. This study comprehensively characterizes the *ORA* gene family in mandarin fish, examining its phylogeny, synteny, gene structure, and selection pressure. Furthermore, we found that each *ORA* displays a distinct expression pattern across multiple tissues, with notable sexual dimorphism, and shows potential binding interactions with specific bile acids and pheromones. Our findings provide valuable insights that enhance the overall understanding of fish ORAs and their potential functions.

## 1. Introduction

Vertebrates rely on their olfactory system to distinguish numerous chemical signals in their environment, which mediates various social behaviors, including food detection, predator avoidance, kin recognition, migration, and reproduction [1,2]. Most terrestrial vertebrates have two distinct olfactory systems: the main olfactory system (MOS) and the vomeronasal system (VNS) [3]. These systems involve four major gene families: olfactory receptors (*OR*s) and trace amine-associated receptors (*TAAR*s), which are expressed in the olfactory epithelium, and the *V1R*s and *V2R*s, which are localized in the vomeronasal organ [4,5,6,7]. Nevertheless, teleost lack a vomeronasal organ and all four types of olfactory receptors are expressed in the olfactory rosette within the fish’s nasal cavity [8]. Traditionally, the MOS has been associated with the detection of environmental odor molecules, and the VNS primarily detects pheromones [9,10].

The V1R-like receptors in fish were initially named based on the mammalian nomenclature, but it has been proposed to rename these *V1R*-like genes as *ORA* (ORs related to class A GPCRs) because fish do not have a vomeronasal organ [11,12]. The origin of the *ORA* gene family is quite ancient, with *V1R*-like genes present in jawless vertebrates such as lampreys. However, it is notable that *ORA*s in teleost and mammals have undergone significant evolutionary divergence. In mammals, the *V1R* family, which originated from a single gene *ORA1* within the *ORA* gene family, shows dynamic evolution with frequent gene gains and losses. The number of *V1R* genes in mammals ranges from 0 to nearly 300. Conversely, teleosts have a relatively small *ORA* gene family, typically with just six members, and this family is highly conserved across species with the exception of a small number of fish with amplifications or deletions in the *ORA* gene [13,14].

Similar to other GPCRs, *ORA*s are seven-transmembrane receptors and maintain the classic three-dimensional structure of Class A GPCRs. In this family, the seven-transmembrane bundle features a traditional ligand-binding pocket that extends from the extracellular membrane boundary [15]. Although the ligands for ORA proteins remain unclear, the conserved features of *ORA* genes in teleosts suggest that this receptor family may detect evolutionarily conserved ligands, such as reproductive pheromones. Some observations support the involvement of *ORA*s in reproductive behaviors, particularly in recognizing sexual pheromones [16]. For instance, *ORA4* in zebrafish (*Danio rerio*) is expressed in the vomeronasal neurons, which have been shown to play a role in fish reproductive behavior [17,18]. *ORA1* in zebrafish has been demonstrated to recognize 4-hydroxyphenylacetic acid, a putative reproductive pheromone that increases spawning frequency in zebrafish pairs, with high specificity and sensitivity [19]. Furthermore, there is evidence that six ORA proteins in zebrafish respond to certain bile acids/salts, which share a common steroid ring structure, although each ORA protein varies in its response to these bile acids/salts [20]. However, it remains unknown whether steroid-sensing receptors in fish are also part of the V1R family.

The mandarin fish (*Siniperca chuatsi*) is widely distributed across China, North Korea, South Korea, and Russia, and it is extensively farmed in China and can be artificially propagated. The ORAs play a crucial role in the breeding behavior of fish, presenting significant potential for applications in artificial breeding [12]. However, there is limited research on the *ORA* in mandarin fish, and the members of the *ORA* gene family and their expression patterns are not yet well understood. In this study, we identified the *ORA* gene family within the mandarin fish genome and conducted an analysis of its gene structure, conserved motifs, and phylogenetic relationships. Furthermore, we examined the expression patterns of these genes in male and female mandarin fish and predicted the recognition of bile acids of *ORA* family genes. Our findings provide a foundational understanding of the biological functions of ORAs in mandarin fish and offer valuable insights for its application in artificial breeding.

## 2. Materials and Methods

### 2.1. Genome-Wide Identification and Physicochemical Properties Analysis of ORA Genes

The complete genome and annotation files for the mandarin fish were downloaded from NCBI (https://www.ncbi.nlm.nih.gov/datasets/genome/GCF_020085105.1/; 15 May 2024). Using fourteen *ORA* sequences (seven from *D. rerio* and seven from medaka (*Oryzias latipes*) as query sequences, potential *ORA* family members were identified through BLAST. Functional annotation was performed via conserved structural analysis of candidate sequences using the online tools Pfam (http://pfam.xfam.org/; 18 May 2024) and SMART (https://smart.embl.de/; 18 May 2024), allowing for the removal of sequences that did not conform to ORA structural characteristics and the identification of the *ORA* family sequences in mandarin fish. The protein structure of ORA was analyzed using the ExPASy-ProtParam online tool (https://web.expasy.org/protparam/; 20 May 2024) to evaluate its physicochemical properties. The subcellular localization of the ORA protein was predicted using WoLF PSORT II (https://wolfpsort.hgc.jp/; accessed on 20 May 2024).

### 2.2. Sequence and Phylogenetic Analysis of ORAs

We collected and identified a total of 108 *ORA* genes from mandarin fish and 17 other teleosts (Supplementary File S1), comparing their similarities. This included 12 species from Percomorpha (*S. chuatsi*, *Centroberyx gerrardi*, *Lampris incognitus*, *Chelon auratus*, *Mastacembelus armatus*, *Clinocottus analis*, *Paralichthys olivaceus*, *Takifugu rubripes*, *Tetraodon nigroviridis*, *Gasterosteus aculeatus*, and *Oreochromis niloticus*), 2 species from Cyprinomorpha (*D. rerio* and *Astyanax mexicanus*), 2 species from Atherinomorpha (*O. latipes* and *Xiphophorus maculatus*), 1 species from Chondrostei (*Lepisosteus oculatus*), 1 species from Clupeomorpha (*Salmo salar*), and 1 species from Parapercomorpha (*Gadus morhua*). Using MEGA-X (Version 10.2.6), we constructed a phylogenetic tree employing the Jones–Taylor–Thornton model (JTT) with 1000 bootstrap replicates. Additionally, we calculated the Ka/Ks (dN/dS) ratios for *ORA*s using MEGA-X to assess the selective pressure acting on these genes. We identified the conserved motifs of ORA proteins in mandarin fish using MEME (http://meme-suite.org/tools/meme; 25 May 2024). Furthermore, we analyzed the collinear patterns of homologous gene blocks among the genomes of mandarin fish, zebrafish, and medaka using the MCScanX toolkit of TBtools (v2.153) with default parameters, visualizing the collinear arrangements of the *ORA* genes using Adobe Illustrator software(Version 2024) [21].

### 2.3. Sample Collections, Total RNA Extraction and Reverse Transcription

The mandarin fish used in this study was cultivated at the Chinese Perch Research Center, Huazhong Agricultural University (Wuhan, China). Before the experiment began, the one-year-old mandarin fish underwent a 12-h acclimation period. Prior to sampling, all mandarin fish were anesthetized using MS-222 (100 mg/L) (Ethyl 3-aminobenzoate methanesulfonate salt, Sigma-Aldrich, St. Louis, MO, USA, A5040). After dissection and sample collection, the samples (lips, eyes, olfactory epithelium, olfactory bulb, gills, brain, skin, muscle, heart, head kidney, trunk kidney, liver, spleen, stomach, intestine, and gonads) were stored at −80 °C for subsequent RNA extraction. This study was approved by the Animal Experiment Ethics Committee of Huazhong Agricultural University (Wuhan, Hubei, China; approval number: HZAUFI-2019-038). Total RNA from each tissue (three samples each) was extracted using the RNAiso Plus Kit (Takara, Beijing, China, code: 9109) according to the manufacturer’s instructions. After evaluating the RNA quality via 1% agarose gel electrophoresis, total RNA was reverse transcribed into cDNA using the PrimeScript RT Reagent Kit with gDNA Eraser (TaKaRa, code: RR047A) [22].

### 2.4. qRT–PCR

Gene expression analysis was performed on each sample using TB Green^®^ Premix Ex Taq™ II (Tli RNaseH Plus) reagent kit (TaKaRa, RR820A). Primers used for qRT–PCR are listed in Supplementary Table S1. The relative expression levels of different samples were compared using the 2^−ΔΔCt^ method [23]. The data are presented as the mean ± standard deviation (S.D.) of four independent replicates. Statistical analysis was performed using one-way analysis of variance (ANOVA), followed by Student’s *t*-test, employing GraphPad Prism software (version 9.0). The significance level was set at *p* < 0.05 (indicating significant) and *p* < 0.01 (indicating highly significant) to determine statistical significance.

### 2.5. Histology Analysis

The olfactory epithelium tissues of 1-year-old mandarin fish were fixed in 4% paraformaldehyde (Beyotime Biotechnology, Shanghai, China, P0099) for 24 h. After that, the samples were dehydrated through a series of graded ethanol solutions (70–100%) (Hushi, Shanghai, China, 10009218), embedded in paraffin, and sectioned at 5 μm thickness. These sections were stained with hematoxylin and eosin stain, then analyzed and photographed using an Olympus BX53 Upright microscope (Olympus, Beijing, China).

### 2.6. Fluorescence In Situ Hybridization (FISH)

Methods of experimentation based on previous literature [24,25], we designed specific probes (CY5-*ORA*) for fluorescent in situ hybridization of each *ORA* and confirmed their specificity by performing a BLAST search against the transcriptome database on NCBI. Four pairs of sequences were designed for each gene and produced by Beijing Tsingke Biotech Co., Ltd. (Tsingke, Beijing, China) (all probe sequences are listed in Supplementary Table S1). The probes were phosphorylated using T4 polynucleotide kinase (Vazyme, Nanjing, China, N102-01) prior to use. All mandarin fish were anesthetized with MS-222 (100 mg/L). After dissection of the nasal cavity, the olfactory epithelium was removed, washed in PBS (DNase, RNase & Protease free, Sterile, Beyotime, ST477), and fixed in 4% paraformaldehyde (Beyotime, P0099) at 4 °C for 24 h. The tissue was then cryoprotected in PBS containing 30% sucrose (Beyotime, P0149D) at 4 °C. The cryoprotected olfactory epithelium was embedded in Tissue-Tek^®^ O.C.T. compound (Sakura Finetek, Tokyo, Japan, 4583), and 10 μm sections were cut using a Leica cryostat (Leica CM3050 S, Wetzlar, Germany). All olfactory epithelium sections were stored at −80 °C.

The sections were sealed in a hybridization chamber and washed three times (5 min each) with PBSTR (0.1% Tween-20 (Beyotime, ST825) and 0.1 U/µL SUPERase•In RNase (Thermo Fisher Scientific, Waltham, MA, USA, AM2694) in PBS). They were then pretreated by fixation (4% PFA) and permeabilization (15 min in 100% methanol at −80 °C followed by 30 min at room temperature with 20 μg/mL proteinase K (Beyotime, ST532)). The hybridization buffer (2× SSC (DNase, RNase & Protease free, Sterile, Beyotime, ST463), 10% formamide (Sigma-Aldrich, MO, USA, F9037), 1% Tween-20, 20 mM RVC (Beyotime, R0107), 0.1 mg/mL salmon sperm DNA (Thermo Fisher Scientific, 15632011), and probes at 100 nM per oligo) was applied to the pretreated olfactory epithelium sections and incubated at 37 °C for 16 h. After hybridization, the samples were washed twice for 20 min each in 4× SSC with PBSTR at 37 °C. The samples were then incubated at room temperature for 2 h with a T4 DNA ligase mixture (1:50 dilution of T4 DNA ligase (Vazyme, C301-01), 1× BSA (Thermo Fisher Scientific, 37525), and 0.2 U/µL SUPERase•In™ RNase) and washed twice with PBSTR. The samples were then incubated at 30 °C for 8 h with an RCA mixture (1:50 dilution of Phi29 DNA polymerase (Vazyme, N106-01), 250 µM dNTPs (Vazyme, P031-01), 1× BSA, and 0.2 U/µL SUPERase-In RNase). Following this, the samples were washed three times for 5 min each with PBSTR. CY5 fluorescent probes (100 nM in 2× SSC) (probes were produced by Sangon Biotech (Shanghai) Co., Ltd., Shanghai, China) complementary to the target were added to the hybridization chamber and incubated at 37 °C for 4 h. After three washes with PBST (PBS with 0.1% Tween-20), the samples were stained with DAPI (Sigma-Aldrich, D9542). Imaging was performed using a Nikon AX/AX R Confocal Microscope System (Tokyo, Japan) with a ×60 oil immersion objective lens.

### 2.7. 3D Structure Modeling and Molecular Docking

AlphaFold 3 (https://alphafoldserver.com; 7 June 2024) was utilized to predict the 3D structure of the mandarin fish ORA protein [26]. The chemical structures of seven bile acids were downloaded from the PubChem website (https://pubchem.ncbi.nlm.nih.gov/, 1 June 2024): lithocholic acid (LCA, 9903), glycolithocholic acid (GLCA, 115245), cholic acid (CA, 221493), taurolithocholic acid (TLCA, 439763), 3-ketolithocholic acid (3-KLCA, 5283906), 7-ketolithocholic acid (7-KLCA, 444262), and 12-ketolithocholic acid (12-KLCA, 3080612) [20]. Molecular docking between five ORAs and seven bile acids was performed in a semi-flexible manner using AutoDock 4, through the Lamarckian Genetic Algorithm 4.2 (Lamarckian GA 4.2), with a total of 50 docking runs. A comprehensive analysis of all the results was conducted. Finally, the docking results were visualized using PyMOL(Version 3.1.0). Similarly, we also examined the binding interactions between ORA and four pheromones: 4-hydroxyphenylacetic acid (4-HCA, 127), prostaglandin F2α (PGF2α, 5280363), androstenedione (AED, 6128), and 17α,20β-dihydroxyprogesterone (17-DHP, 107701).

## 3. Results

### 3.1. Five ORAs of Mandarin Fish Are Localized on Two Different Chromosomes

**Figure 1:**
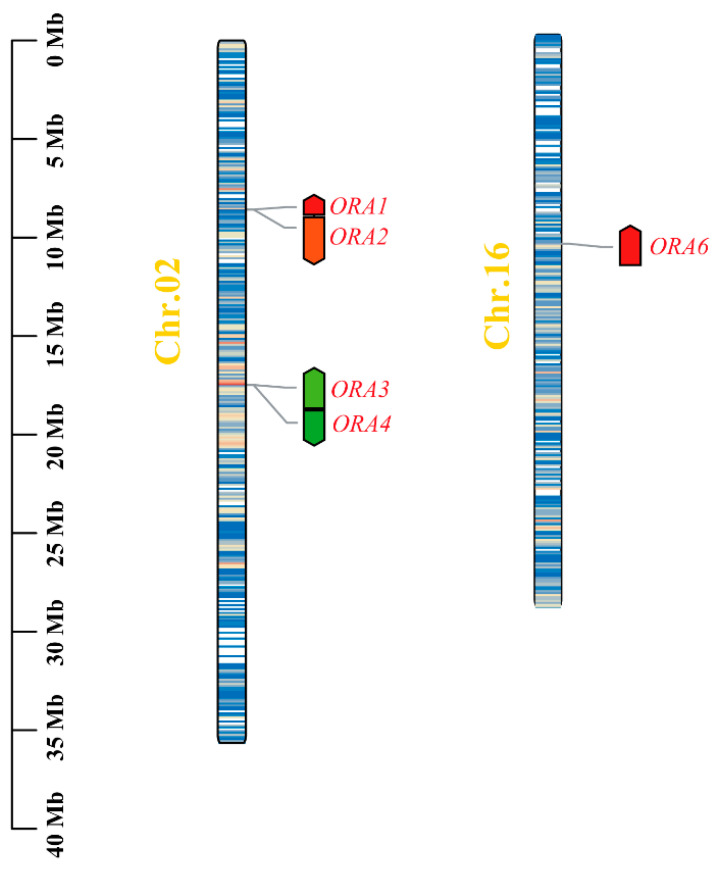
Chromosomal localization of *ORA* members in mandarin fish. *ORA1*, *ORA2*, *ORA3*, and *ORA4* are located on chromosome 2, while *ORA6* is located on chromosome 16.

To identify the members of the ORA family in mandarin fish, we compared 14 *ORA* sequences from zebrafish and medaka with the mandarin fish genome (PRJNA767607) [27]. Through sequence similarity and conserved domain analysis, we identified a total of five potential *ORA* genes (GenBank accession numbers: PQ662472, PQ662473, PQ662474, PQ662475, and PQ662476). These *ORA* genes are distributed across two different chromosomes: *ORA1*, *ORA2*, *ORA3*, and *ORA4* are located on chromosome 2, while *ORA6* is found on chromosome 16 (Figure 1, Supplementary Table S2). To further explore the physicochemical properties of the ORA proteins, we conducted a bioinformatics assessment (Table 1). The results revealed that the number of amino acids in the ORA proteins ranges from 307 to 340, with molecular weights varying between 34.12 and 37.39 kDa. The theoretical isoelectric points (pI) of the mandarin fish ORAs range from 8.40 to 10.34, indicating that they are basic proteins. The instability indices of ORA1, ORA3, and ORA6 are all below 40, suggesting that these proteins are relatively stable. Furthermore, the aliphatic index of all ORA members exceeds 100, and the grand average of hydropathicity (GRAVY) is greater than 0, indicating that the ORAs are hydrophobic proteins. Predicted subcellular localization results suggest that the ORAs are located in the plasmalemma.

### 3.2. ORAs Are Highly Conserved in Evolution

**Figure 2:**
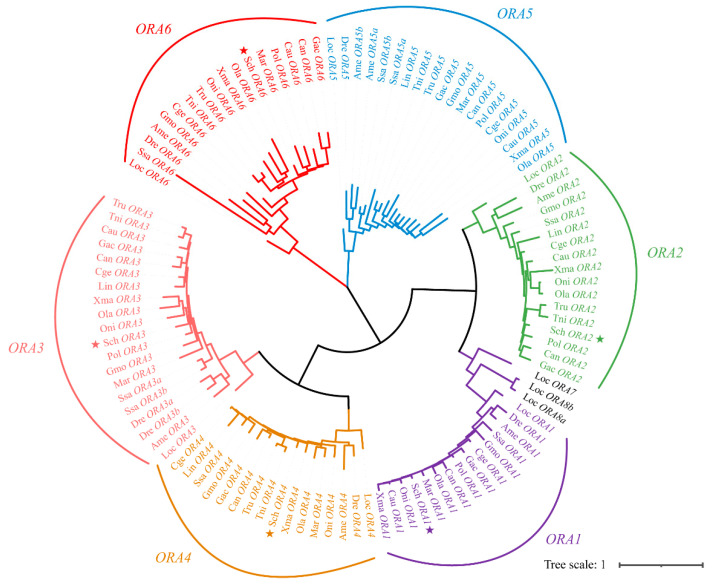
Phylogenetic tree of *ORA* family in teleosts. Phylogenetic tree of 108 *ORA* sequences from 18 teleosts was constructed using maximum likelihood method, with 1000 bootstrap replicates. Sch, *S. chuatsi*; Cge, *C. gerrardi*; Lin, *L. incognitus*; Cau, *C. auratus*; Mar, *M. armatus*; Can, *C. analis*; Pol, *P. olivaceus*; Tru, *T. rubripes*; Tni, *T. nigroviridis*; Gac, *G. aculeatus*; Oni, *O. niloticus*; Dre, *D. rerio*; Ame, *A. mexicanus*; Ola, *O. latipes*; Xma, *X. maculatus*; Loc, *L. oculatus*; Ssc, *S. salar*; Gmo, *G. morhua*. ★ represent *ORA*s identified in *S. chuatsi*.

**Figure 3:**
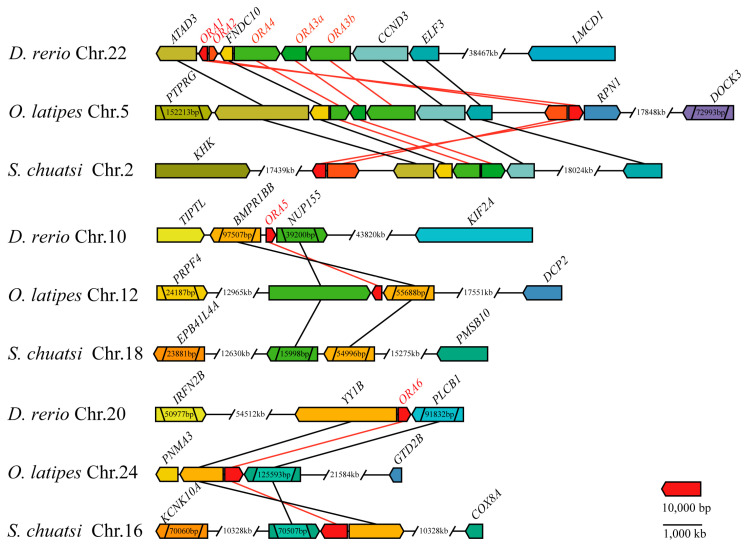
Collinearity analysis of *ORA*s. Inter-species collinearity analysis of *ORA* genes of *D. rerio*, *O. latipes* and *S. chuatsi*. Arrows representing genes indicate coding strands, while red lines illustrate collinearity relationships between *ORA*s.

To more accurately predict the function of ORAs and further investigate the evolutionary relationships of ORA proteins among different species, a phylogenetic tree was constructed using the 108 *ORA* sequences of mandarin fish and 17 teleosts (Figure 2). Based on phylogenetic relationships, all *ORA*s were categorized into six subtypes. Mandarin fish retained five subtypes: *ORA1*, *ORA2*, *ORA3*, *ORA4*, and *ORA6*, while *ORA5* is absent. Collinearity analysis was performed using MCScanX to compare the genomes of mandarin fish with two model species, zebrafish and medaka (Figure 3). By examining the genomic regions flanking *ORA*s on zebrafish chromosomes 10, 20, and 22, medaka chromosomes 5, 12, and 24, and mandarin fish chromosomes 2 and 16, the conservation of homologous genes was investigated. The syntenic analysis revealed that the *ORA3*, *ORA4*, and *ORA6* loci are highly conserved among zebrafish, medaka, and mandarin fish. Zebrafish and medaka each possess two copies of the *ORA3* gene, whereas mandarin fish has only one. These genes are located between fibronectin type III domain containing 10 (*FNDC10*) and Cyclin D3 (*CCND3*). *ORA6* is situated between Yin Yang 1B (*YY1B*) and phospholipase C beta 1 (*PLCB1*). In contrast, *ORA1* and *ORA2* are not conserved across the loci in the three fishes.

### 3.3. ORAs Maintain a Conserved Exon–Intron Structure Typical of Teleosts

**Figure 4:**
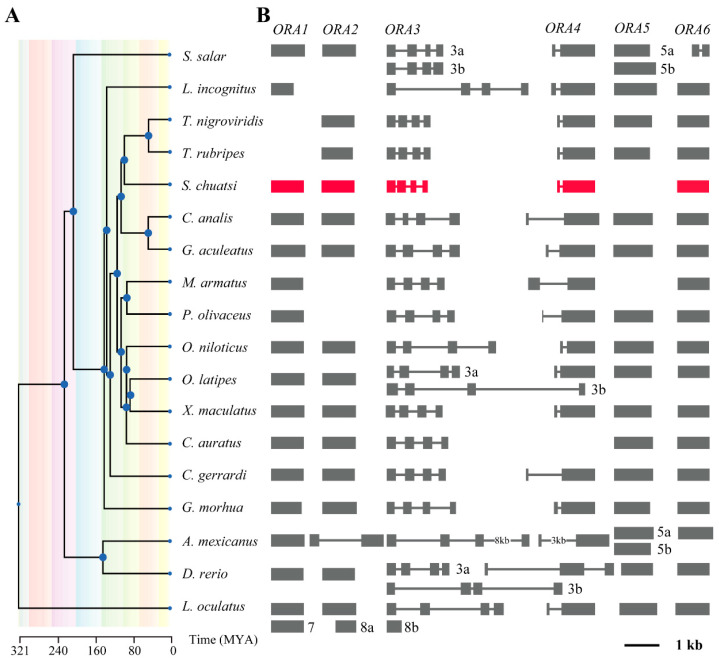
Conserved structural of *ORA* genes in teleosts. (**A**) Evolutionary tree of 18 teleosts from Timetree. Different colors represent different geological periods. (**B**) Structure of *ORA*s includes exons and introns.

We examined the gene structures of *ORA*s from 18 species of teleosts for further analyzing the conservation features of the *ORA* family (Figure 4). The results indicated that *ORA1*, *ORA2*, *ORA5*, and *ORA6* exist as single-exon genes across teleosts, except for *ORA2* in *A. mexicanus* and *ORA6* in *S. salar*, and some teleosts exhibit partial loss of *ORA* genes. In contrast, *ORA3* is composed of four exons, while *ORA4* consists of two exons, with relatively consistent lengths in corresponding exons, although there is significant variation in intron lengths. These findings demonstrate a high degree of structural conservation of *ORA*s among teleosts.

### 3.4. Mandarin Fish ORAs Have Conserved Motifs

**Figure 5:**
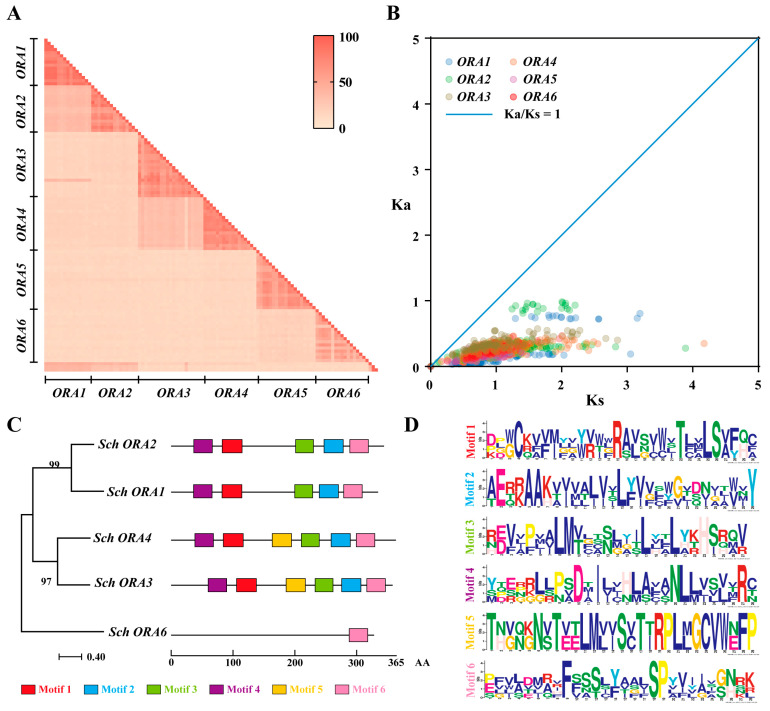
Conservation Analysis of *ORA*s. (**A**) Similarity analysis of amino acids from *ORA*s of 18 species of teleost. (**B**) Analysis of selective pressure on *ORA*s. Values of Ka/Ks less than 1 indicate that *ORA*s in fish have undergone purifying selection during evolution. (**C**) Phylogenetic tree of *ORA*s from mandarin fish and motif analysis. (**D**) Colored boxes represent different conserved motifs, with conserved sequence displayed on far right.

Furthermore, we compared the amino acid similarity of ORAs across the 18 teleosts (Figure 5A, Supplementary Table S3). The similarity between ORA subtypes of the same species was notably high, exceeding 50% even among different orders, while similarity among *ORA* subtypes within the same order was over 70%. However, the similarity between different *ORA* subtypes was low, even within the same species (less than 40%). We calculated the nonsynonymous (Ka) and synonymous (Ks) substitution rates (Ka/Ks) for the coding sequences (CDS) of *ORA*s from teleost (Figure 5B, Supplementary Table S4). The results showed that the Ka/Ks values for *ORA*s were less than 1, indicating that *ORA*s in teleost have undergone purifying selection during evolution. Using the MEME online software, we analyzed the composition and number of conserved motifs in mandarin fish *ORA*s, identifying six conserved motifs, labeled as motif1 to motif 6 (Figure 5C,D). Notably, *ORA6* in mandarin fish contains only motif 6, suggesting that the conserved motifs in *ORA6* have undergone significant changes during evolution.

### 3.5. ORAs Exhibit Distinct Expression Profiles in Various Tissues with Notable Sexual Dimorphism

**Figure 6:**
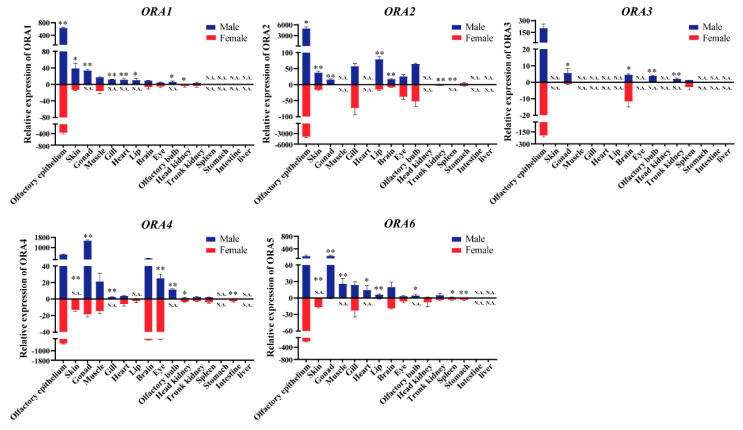
Tissue expression profile of *ORA* family in *S. chuatsi*. RT-qPCR analysis shows expression levels of *ORA1*, *ORA2*, *ORA3*, *ORA4*, and *ORA6* across 16 tissues in both male and female *S. chuatsi*, along with sex-based expression differences. Blue represents males, and red represents females. * indicates *p* < 0.05 (significant), and ** indicates *p* < 0.01 (highly significant). N.A. denotes no detectable expression.

To investigate the functional tissues of *ORA* genes, we performed qRT-PCR to analyze the expression levels of these five genes across 16 different tissues in mandarin fish: lips, eyes, olfactory epithelium, olfactory bulb, gills, brain, skin, muscle, heart, head kidney, trunk kidney, liver, spleen, stomach, intestine, and gonads (Figure 6). Furthermore, we compared the expression differences between male and female individuals. Each ORA exhibits a unique expression profile, with notable sexual dimorphism in certain tissues. We detected the expression of ORAs from either females or males or both in 15 different tissues, with no expression observed in the liver. Most tissues showed only low levels of expression. ORA1 and ORA3 were highly expressed exclusively in the olfactory epithelium (in both males and females). ORA2 exhibited high expression in the olfactory epithelium (both males and females), gills (both males and females), lips (males), and olfactory bulb (both males and females). ORA4 showed relatively high expression in the olfactory epithelium (both males and females), gonads (male), brain (both males and females), and eyes (both males and females). ORA6 was highly expressed only in the olfactory epithelium (both males and females) and gonads (males). Notably, only the olfactory epithelium and brain exhibited detectable levels of all five ORAs in both males and females. ORA1 and ORA2 showed sex differences in expression within the olfactory epithelium, while ORA3 displayed sex differences in the brain. Additionally, all ORAs were expressed in the male gonads, with significantly higher expression compared to the female gonads (ORA1 was not detected in the female gonads).

### 3.6. ORAs Are Expressed in Microvillus Olfactory Receptor Neurons or Crypt Cells in the Olfactory Epithelium

**Figure 7:**
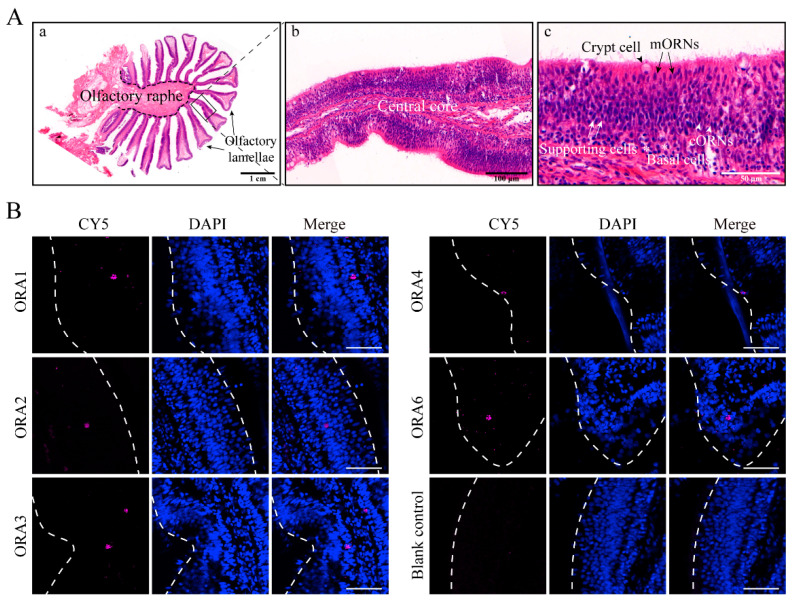
Localization of ORAs in olfactory epithelium. (**A**) H.E. staining of olfactory rosette in grouper. In panel a, the black dashed line indicates the olfactory raphe, and the black arrow points to the olfactory lamellaes. Panel b shows the olfactory lamellae, with the central core in the middle of the olfactory raphe. In panel c, the black short arrow points to crypt cells, the black long arrow points to microvillus olfactory receptor neurons (mORNs), the white short arrow points to ciliated olfactory receptor neurons (cORNs), the white long arrow points to supporting cells, and the asterisk (*) represents basal cells. (**B**) Fluorescence in situ hybridization was used to identify cells expressing *ORA1*, *ORA2*, *ORA3*, *ORA4*, and *ORA6* in olfactory epithelium in *S. chuatsi*. Red represents cells expressing *ORA*s, and blue indicates cell nuclei stained with DAPI. Scale bar is 50 μm. The white dashed line indicates the boundary of the olfactory lamellae.

In teleosts, olfactory cells are classified into three types: ciliated olfactory receptor neurons (cORNs), microvillus olfactory receptor neurons (mORNs), crypt cells (CCs), and kappe neurons (κNs) each serving distinct functions [28]. We identified three neuronal sections by H.E. staining (Figure 7A). To gain a better understanding of the role of *ORA*s, we investigated the specific cell types that express *ORA*s. Using fluorescence in situ hybridization, we mapped the distribution of *ORA*s in olfactory tissues (Figure 7B). The results revealed that *ORA1*, *ORA2*, *ORA3*, and *ORA6* are expressed in the middle layers of the olfactory lamella, suggesting that these four *ORA*s are localized to mORNs. In contrast, *ORA4* is expressed in the out layer of the olfactory lamella, which likely corresponds to crypt cells.

### 3.7. ORAs Exhibit an Affinity for Bile Acids and Sex Pheromones

**Figure 8:**
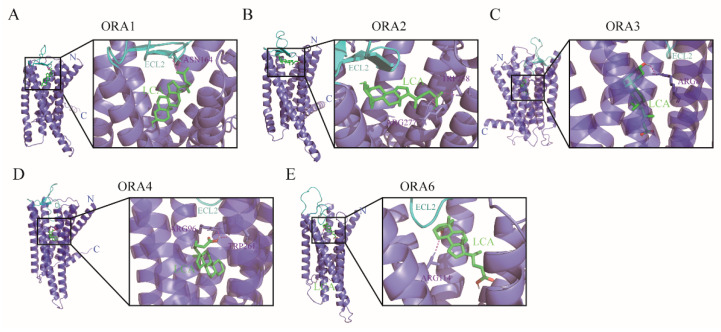
Molecular docking of ORA proteins with bile acids. Autodock4 was used to predict binding sites of *S. chuatsi* ORA1 (**A**), ORA2 (**B**), ORA3 (**C**), ORA4 (**D**), and ORA6 (**E**) with LCA. Green molecules represent respective bile acids, and red dashed lines indicate hydrogen bonds. ECL2 is extracellular loop of cell 2.

**Figure 9:**
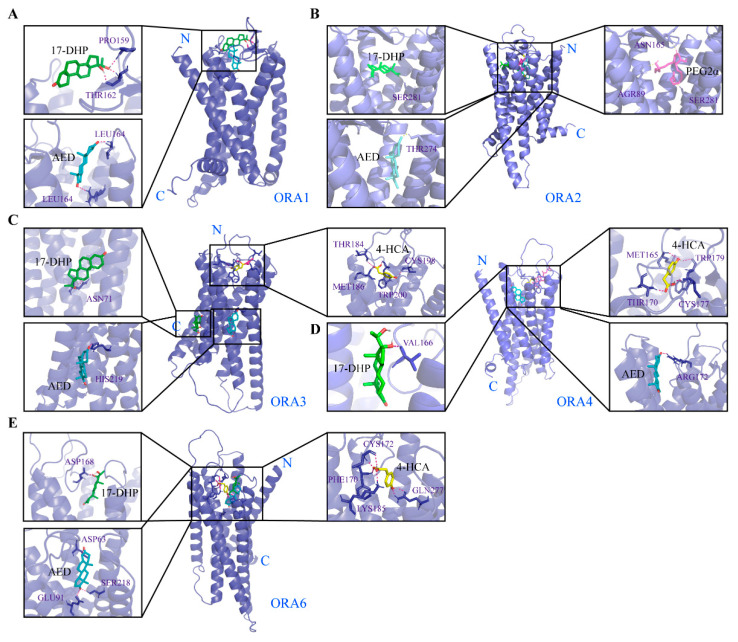
Binding site prediction of mandarin fish ORAs with four pheromones. Autodock4 was used to predict binding sites of *S. chuatsi* ORA1 (**A**), ORA2 (**B**), ORA3 (**C**), ORA4 (**D**), and ORA6 (**E**) with four pheromones: 4-HCA, 17-DHP, AED, and PGF2α. Green molecules represent 17-DHP, blue molecules represent AED, pink molecules represent PGF2α, and yellow molecules represent 4-HCA. Red dashed lines indicate hydrogen bonds.

ORAs have been reported to recognize bile salts in zebrafish, prompting us to predict the binding potential of the five ORAs in mandarin fish with seven types of bile salts (lithocholic acid, LAC; glycolithocholic acid, GLAC; cholic acid, CA; taurolithocholic acid, TLCA; 3-ketolithocholic acid, 3-KLCA; 7-ketolithocholic acid, 7-KLCA and 12-ketolithocholic acid, 12-KLCA). First, we used AlphaFold 3 to model the 3D structures of the five ORA proteins in mandarin fish, obtaining reliable models (pTM > 0.8) (Figures S1 and S2). Subsequently, we employed Autodock4 to perform molecular docking simulations between the ORA proteins and bile acids. We selected the five lowest binding energy values for each ORA with the bile salts for comparison (Table 2). The results show that the binding energies of the five ORAs with seven bile acids are all below −5 kcal/mol, indicating a strong binding potential between the ORAs and these bile salts. Among them, ORA1, ORA3, and ORA6 display the lowest binding energies with LCA, while ORA2 and ORA4 exhibit the lowest binding energies with CA. Additionally, we investigated the binding sites of each ORA with the bile salt that exhibited the lowest binding energy. The prediction results reveal that the binding sites of the ORAs with these bile salts are primarily located in the extracellular domains (Figure 8A–E). We also examined the binding potential of ORAs with four signaling molecules: 4-hydroxyphenylacetic acid (4-HCA), prostaglandin F2α (PGF2α), androstenedione (AED), and 17α, 20β-dihydroxyprogesterone (17-DHP) (Table 3). The minimum binding energies of ORAs with 17-DHP and AED are relatively low, below −5 kcal/mol. Except for ORA2, which has a minimum binding energy of −6.1 kcal/mol with PGF2α, the binding energies of the other ORAs with PGF2α are all above −5 kcal/mol. Additionally, ORA3, ORA4, and ORA6 exhibit relatively low binding energies with 4-HCA. Likewise, we present the binding sites of ORAs with these signaling molecules, where the binding energies are below −5 kcal/mol (Figure 9A–E).

## 4. Discussion

**Figure 10:**
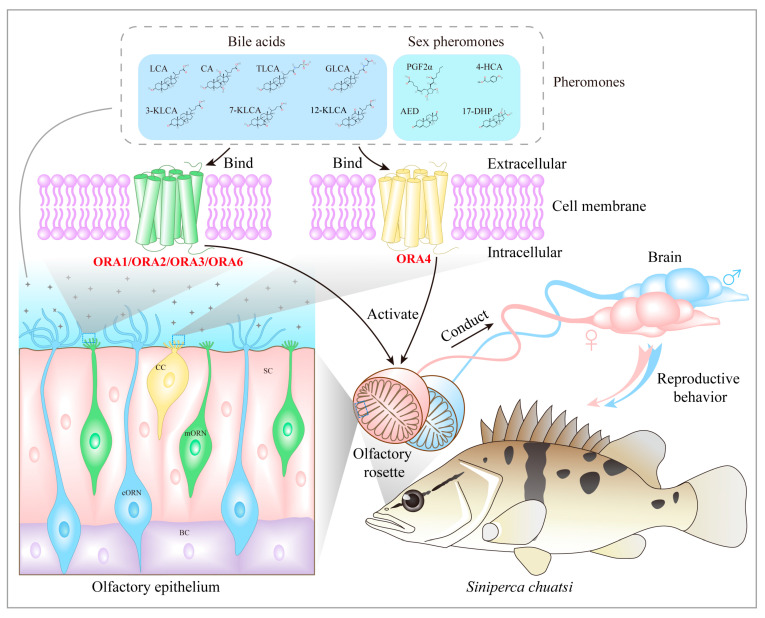
Characteristics of olfactory system in mandarin fish and *ORA* family expressed in olfactory epithelium. Female or male mandarin fish express *ORA*s in the microvillus olfactory receptor neurons (mORNs) or crypt cells (CCs) of the olfactory epithelium, and after recognizing bile acids or sex pheromones in the aquatic environment, the information is transmitted to the brain, triggering reproductive behaviors in males or females.

*ORA*s are an ancient and conserved class of olfactory receptors in fish that play a crucial role in various vital processes. With advancements in sequencing technology, the identification of *ORA*s in fish has progressed significantly. In this study, we aimed to characterize the *ORA* gene members from the mandarin fish genome to enhance our understanding of teleost *ORA*s. We identified five *ORA*s: *ORA1*, *ORA2*, *ORA3*, *ORA4*, and *ORA6*, and we analyzed the gene structure, conservation, and expression patterns of the mandarin fish *ORA*s and identified the cell types that express *ORA*s in the olfactory epithelium (Figure 10).

Unlike *OR*s, *V2R*s, and *TAAR*s, which have undergone significant amplification in fish genomes, the number of *ORA*s is generally more conserved across most teleosts, typically numbering around six [13,14,29,30,31,32,33]. However, we found that *ORA5* is absent in the mandarin fish genome. In fact, instances of *ORA* gene loss or expansion have also been observed in certain teleosts; for example, *T. rubripes* and *T. nigroviridis* lack ORA1, *L. incognitus* and *P. olivaceus* are missing *ORA2*, and *C. auratus* lacks *ORA4*. Additionally, *M. armatus* is missing both *ORA2* and *ORA4*. Expansions in the *ORA* family have been noted in zebrafish (*ORA3a*/*b*), medaka (*ORA3a*/*b*), *S. salar* (*ORA3a*/*3b*, *ORA5a*/*b*), and *X. maculatus* (*ORA5a/5b*) [34,35]. Notably, all instances of gene loss among the 18 teleosts we studied occurred within the Percomorpha clade. However, this loss does not exhibit a clear pattern, and the underlying reasons for this phenomenon remain unclear. Despite the absence of *ORA5* in mandarin fish, the remaining five *ORA*s still exhibit the ancient and conserved characteristics typical of this receptor family. They share a similar number of exons and demonstrate high sequence similarity with *ORA*s in other teleosts [13]. Although the positions of *ORA1* and *ORA2* have shifted on the chromosomes, the loci of *ORA3*, *ORA4*, and *ORA6* are highly consistent among zebrafish, medaka, and mandarin fish. In addition, the *ORA* family received strong purification selection pressure in different teleosts. These evidences further support the likelihood of functional conservation for *ORA*s.

We detected abundant expression of five ORAs in the olfactory organs of the mandarin fish, which aligns with our expectations. However, we also observed their widespread expression in various other tissues. In fact, ectopic expression of olfactory receptors (*OR*s, *VR*s, and *TAAR*s) is a common phenomenon, both in mammals and in teleost fish [8,36,37]. The ectopic expression of olfactory receptors suggests they may serve diverse biological functions. *OR*s have been implicated in processes such as sperm chemotaxis, wound healing, and muscle regeneration, while *TAAR*s are known to influence food intake and regulate blood glucose levels [38,39,40,41,42]. The underlying cause of the ectopic expression of *ORA*s remains unclear. However, we have observed that *ORA*s are expressed in organs such as fish gills and skin, which are directly exposed to the aquatic environment. This may enhance the fish’s ability to detect pheromones in the water. Furthermore, the expression of *ORA*s in internal organs suggests that they may also play a role in recognizing endogenous ligands, such as bile acids, and regulating associated biological processes. Furthermore, mandarin fish *ORA*s exhibit variable expression levels in the gonads (testes and ovaries), showing sexual dimorphism, which is worth noting. *ORA*s have traditionally been considered pheromone receptors, and the sexual dimorphism of *ORA*s in the gonads of mandarin fish suggests that their functions may differ between males and females, a hypothesis that warrants further investigation.

In the olfactory epithelium of teleost fish, four types of olfactory neurons exist: cORNs, mORNs, CCs, and kNs. The mORNs are typically located in the superficial layer, while cORNs are found in the deeper layers. Mature CCs are located in the out layer, with kNs situated at the very surface [28,43]. In zebrafish, *ORA1* is expressed in mORNs, *ORA4* in CCs, while the expression of other *ORA*s remains undetermined [17]. In mandarin fish, we found that *ORA1*, *ORA2*, *ORA3*, and *ORA6* are expressed in the middle layer of the olfactory epithelium, which closely corresponds to the distribution of mORNs, suggesting that these four genes may be expressed in mORNs. *ORA4* is expressed in the outer layer, and based on findings from zebrafish, we hypothesize that mandarin fish ORA4 is also expressed in CCs. A long-standing hypothesis is that different types of ORNs in fish specialize in detecting distinct classes of odorants: cORNs preferentially respond to bile salts, mORNs are more attuned to amino acids and nucleotides, and CCs play a role in detecting olfactory stimuli related to reproductive behavior [28,44,45]. However, a recent study elegantly demonstrated that zebrafish ORAs respond to bile salts, despite not being expressed in cORNs [20]. ORAs have traditionally been considered conserved receptors for pheromones due to their structural conservation, but direct evidence remains scarce, as ORA ligands have only been identified in zebrafish [10]. Nevertheless, it is still uncertain whether ORAs in other fishes possess similar functions, and the functional conservation of ORAs requires further validation. Based on our predictions regarding the relationship between mandarin fish ORAs and bile salts, the role of ORAs in mandarin fish may be similar to that in zebrafish. Mandarin fish ORAs demonstrate a high affinity for bile salts, suggesting they may also be involved in the recognition of these compounds. Bile salts are effective olfactory stimuli for fish, and their detection has been observed throughout the evolutionary development of fishes [46,47,48]. Bile salts contribute to various behaviors in fish, including detecting nearby conspecifics, foraging, migrating, and spawning [49]. However, mandarin fish do not exhibit migratory behavior, and bile salts as pheromones have only been reported in marine lampreys. We hypothesize that bile salts may assist mandarin fish in locating nearby conspecifics or/and finding food, although this hypothesis needs further validation. Additionally, zebrafish ORA1 recognizes 4-hydroxyphenylacetic acid, which triggers spawning behavior and increases spawning frequency [19]. Our predictive results indicate that mandarin fish ORA1 has some affinity for 4-hydroxyphenylacetic acid, ORA1 binding ability is significantly higher for bile acids. Conversely, ORA1 displays a strong affinity for androstenedione and PGF2α, suggesting a high likelihood of ligand recognition. Similarly, other ORAs in mandarin fish show comparable preferences for these four sex pheromones.

## 5. Conclusions

We identified five ORA genes in mandarin fish, which are distributed across two chromosomes (*ORA1*, *ORA2*, *ORA3*, and *ORA4* are located on chromosome 2, while *ORA6* is located on chromosome 16). Phylogenetic analysis indicates that the ORA family is divided into six subfamilies. Synthesis, gene structure, and selective pressure analyses suggest that the ORAs in mandarin fish have undergone strong purifying selection, exhibiting highly conserved chromosomal locations and gene structures compared to other fish species. Additionally, ORAs show a high degree of sequence similarity and contain six conserved motifs in teleosts. Each ORA exhibits a unique expression pattern across various tissues, with distinct sexual dimorphism. Moreover, we identified the types of olfactory neurons expressing *ORA*s. *ORA1*, *ORA2*, *ORA3*, and *ORA6* are expressed in the microvillar sensory neurons of the olfactory epithelium, while *ORA4* is expressed in crypt cells. We also predicted the potential binding interactions of mandarin fish ORAs with bile acids and specific sex pheromones. Our findings provide valuable insights into the study of fish ORAs and their potential functions. Furthermore, our conclusions may contribute to the application of ORAs in the artificial breeding of mandarin fish.

## Figures and Tables

**Table 1:** Physicochemical properties of ORA proteins in mandarin fish (*Siniperca chuatsi*).

Gene	Exon Number	Open Reading Frame Length (bp)	Number of Amino Acids	Molecular Weight (KDa)	Theoretical pI	Instability Index	Aliphatic Index	Grand Average of Hydropathicity (GRAVY)	Number of Transmembrane Domains	Subcellular Localization
ORA1	1	942	313	34.12	9.27	35.52	116.17	0.707	7	plasmalemma
ORA2	1	969	322	35.15	9.58	40.11	122.27	0.675	7	plasmalemma
ORA3	4	1008	335	37.11	10.34	37.40	118.96	0.593	7	plasmalemma
ORA4	2	1023	340	37.39	8.4	47.32	120.03	0.613	7	plasmalemma
ORA6	1	924	307	34.34	9.08	33.09	120.52	0.702	7	plasmalemma

**Table 2:** Binding energy of *ORA*s with bile acids.

Gene	Bile Salts	Binding Energy (kcal/mol)				
		1st	2nd	3rd	4th	5th
ORA1	3-KLCA	−5.77 (0)	−5.73 (0)	−5.64 (0)	−5.47 (1)	−5.41 (0)
	7-KLCA	−5.38 (1)	−4.96 (2)	−4.85 (0)	−4.80 (1)	−4.78 (0)
	12-KLCA	−5.67 (0)	−5.64 (1)	−5.49 (1)	−5.42 (1)	−5.41 (1)
	CA	−5.23 (1)	−5.09 (0)	−4.94 (0)	−4.76 (0)	−4.73 (0)
	GLCA	−5.54 (1)	−4.84 (1)	−4.09 (0)	−4.08 (0)	−3.87 (1)
	LCA	−5.78 (2)	−5.53 (1)	−5.51 (0)	−5.47 (1)	−5.41 (0)
	TLCA	−5.60 (1)	−5.42 (1)	−5.39 (0)	−4.75 (1)	−4.67 (1)
ORA2	3-KLCA	−5.79 (0)	−5.62 (0)	−5.12 (0)	−4.92 (1)	−4.85 (0)
	7-KLCA	−5.04 (2)	−4.79 (0)	−4.70 (0)	−4.44 (2)	−4.39 (2)
	12-KLCA	−5.35 (1)	−5.09 (0)	−4.97 (0)	−4.97 (1)	−4.88 (0)
	CA	−6.64 (1)	−5.40 (1)	−5.40 (0)	−5.00 (0)	−4.94 (0)
	GLCA	−5.68 (0)	−5.56 (0)	−5.56 (0)	−5.38 (1)	−4.82 (1)
	LCA	−5.74 (1)	−5.34 (1)	−5.23 (0)	−5.07 (1)	−5.06 (1)
	TLCA	−5.32 (1)	−5.06 (2)	−4.73 (3)	−4.48 (0)	−4.19 (1)
ORA3	3-KLCA	−6.77 (0)	−5.63 (0)	−5.48 (1)	−5.43 (1)	−5.31 (0)
	7-KLCA	−6.23 (1)	−5.61 (2)	−5.01 (0)	−4.79 (0)	−4.70 (0)
	12-KLCA	−6.14 (0)	−6.02 (0)	−5.69 (1)	−5.60 (2)	−5.59 (2)
	CA	−6.39 (0)	−5.92 (0)	−5.71 (0)	−5.67 (0)	−5.24 (1)
	GLCA	−5.07 (1)	−4.16 (0)	−4.15 (0)	−4.01 (2)	−4.01 (0)
	LCA	−6.92 (4)	−5.60 (0)	−5.57 (0)	−5.34 (0)	−5.28 (1)
	TLCA	−5.74 (2)	−4.63 (0)	−4.59 (0)	−4.38 (0)	−4.34 (0)
ORA4	3-KLCA	−5.59 (1)	−5.48 (2)	−5.05 (0)	−5.02 (0)	−5.01 (1)
	7-KLCA	−4.58 (1)	−4.29 (0)	−4.08 (1)	−3.93 (0)	−3.92 (1)
	12-KLCA	−5.83 (1)	−5.67 (2)	−5.02 (0)	−5.00 (1)	−4.99 (0)
	CA	−6.29 (1)	−6.19 (0)	−6.19 (1)	−6.08 (0)	−5.64 (1)
	GLCA	−5.85 (1)	−5.11 (0)	−4.92 (0)	−4.71 (1)	−4.39 (1)
	LCA	−5.81 (0)	−5.80 (1)	−5.53 (1)	−5.44 (1)	−5.33 (0)
	TLCA	−4.88 (1)	−4.60 (1)	−4.59 (1)	−4.48 (1)	−4.29 (1)
ORA6	3-KLCA	−6.10 (0)	−5.86 (0)	−5.82 (2)	−5.64 (1)	−5.44 (0)
	7-KLCA	−5.26 (1)	−4.84 (0)	−4.81 (0)	−4.62 (0)	−4.54 (0)
	12-KLCA	−5.22 (1)	−5.19 (0)	−5.12 (2)	−5.06 (1)	−4.90 (2)
	CA	−6.00 (0)	−5.98 (0)	−5.39 (0)	−5.36 (2)	−5.11 (0)
	GLCA	−6.29 (1)	−5.59 (1)	−4.98 (1)	−4.56 (1)	−4.50 (1)
	LCA	−6.82 (1)	−5.72 (2)	−5.50 (1)	−5.48 (1)	−5.38 (2)
	TLCA	−5.13 (1)	−4.60 (0)	−4.42 (1)	−4.25 (1)	−4.12 (0)

Note: Numbers in parentheses represent number of hydrogen bond.

**Table 3:** Binding energy of ORAs with pheromones.

Gene	Pheromones	Binding Energy (kcal/mol)				
		1st	2nd	3rd	4th	5th
ORA1	4-HCA	−3.42 (2)	−3.38 (2)	−3.36 (2)	−3.31 (2)	−3.31 (2)
	17-DHP	−6.49 (2)	−6.13 (3)	−6.06 (0)	−5.81 (0)	−5.80 (0)
	AED	−7.22 (0)	−7.19 (1)	−6.78 (0)	−6.68 (0)	−6.63 (1)
	PGF2α	−1.90 (3)	−1.78 (1)	−1.43 (1)	−1.39 (0)	−1.24 (1)
ORA2	4-HCA	−4.50 (1)	−3.74 (3)	−3.63 (2)	−3.06 (1)	−2.99 (2)
	17-DHP	−5.97 (2)	−5.65 (0)	−5.62 (0)	−5.44 (0)	−5.32 (2)
	AED	−7.76 (0)	−7.58 (0)	−7.23 (0)	−7.11 (0)	−6.95 (1)
	PGF2α	−6.10 (4)	−5.31 (4)	−3.71 (4)	−3.01 (0)	−2.32 (0)
ORA3	4-HCA	−5.24 (2)	−4.86 (1)	−4.66 (2)	−4.59 (2)	−4.52 (2)
	17-DHP	−7.29 (3)	−6.97 (2)	−6.77 (1)	−6.70 (2)	−6.70 (2)
	AED	−9.66 (0)	−9.60 (0)	−7.39 (0)	−7.35 (1)	−7.34 (0)
	PGF2α	−4.61 (3)	−4.34 (3)	−3.42 (1)	−2.88 (0)	−2.46 (2)
ORA4	4-HCA	−6.27 (2)	−6.24 (2)	−6.07 (2)	−6.01 (2)	−5.86 (3)
	17-DHP	−6.94 (1)	−6.90 (1)	−6.89 (1)	−6.88 (3)	−6.86 (0)
	AED	−8.34 (0)	−7.94 (0)	−7.92 (1)	−7.92 (1)	−7.92 (1)
	PGF2α	−4.20 (1)	−3.24 (2)	−3.05 (0)	−2.92 (2)	−2.69 (0)
ORA6	4-HCA	−5.61 (4)	−5.44 (4)	−5.09 (3)	−4.91 (1)	−4.42 (2)
	17-DHP	−7.57 (2)	−7.47 (2)	−7.09 (2)	−7.02 (2)	−6.80 (1)
	AED	−8.52 (1)	−8.52 (1)	−8.51 (1)	−8.47 (1)	−6.95 (0)
	PGF2α	−4.48 (3)	−4.37 (2)	−3.88 (1)	−3.13 (3)	−1.86 (1)

Note: Numbers in parentheses represent number of hydrogen bond.

## Data Availability

All data used in this study are available in the Supplementary Materials or from NCBI.

## Supplementary Materials

The following supporting information can be downloaded at: https://www.mdpi.com/article/10.3390/cells14030189/s1, Supplementary Table S1: Primers and probes used in this study. Supplementary Table S2: Localization of *ORA*s on chromosomes in mandarin fish. Supplementary Table S3: Amino acid similarity of *ORA*s. Supplementary Table S4: Nonsynonymous (Ka) and synonymous (Ks) substitution rates (Ka/Ks) for coding sequences (CDS) of ORAs. Supplementary File S1: Amino acid sequences used to construct phylogenetic tree. Supplementary Figure S1: Predicted template modeling (pTM) score of mandarin fish ORA proteins. Supplementary Figure S2: 3D structures of ORAs predicted by AlphaFold 3.
