# Supplementary material for: Genome-Wide Identification, Characterization of the ORA (Olfactory Receptor Class A) Gene Family, and Potential Roles in Bile Acid and Pheromone Recognition in Mandarin Fish (Siniperca chuatsi)
Source: Cells. 2025 Jan 26;14(3):189. doi: 10.3390/cells14030189 (PMC11817882; doi:10.3390/cells14030189)
Supplement: Supplementary file 1 [file cells-14-00189-s001.zip › Supplementary File.pdf]

Supplementary File S1: Amino acid sequences used to construct phylogenetic tree.

The phylogenetic tree of 108 *ORA* sequences from 18 teleost was constructed using the maximum likelihood method. Sch, *S. chuatsi*; Cge, *C. gerrardi*; Lin, *L. incognitus*; Cau, *C. auratus*; Mar, *M. armatus*; Can, *C. analis*; Pol, *P. olivaceus*; Tru, *T. rubripes*; Tni, *T. nigroviridis*; Gac, *G. aculeatus*; Oni, *O. niloticus*; Dre, *D. rerio*; Ame, *A. mexicanus*; Ola, *O. latipes*; Xma, *X. maculatus*; Loc, *L. oculatus*; Ssc, *S. salar*; Gmo, *G. morhua*.

>Sch_ORA1

MDLCVTIKGVSFLLQTGMGILGNAMVLLAYGLIIYTEPKLLPVDMILCHLAFANLMLL
LTRCVPQTMTVFGLRDLLNDPGCKVVIYAYRIGRALSVCITCMLSVFQAVTIAPAGPRL
SRLKLALPSLVLPTFAALWLLNMAICIAAPFFSMAPRNGTVPAFTLNLGFCHVDFRDN
LSYVINGVAVSGRDFAFVALMVGSSGYILLLLHRHSHQVRGIRRSQGSGAETKAAKTV
VTLVVLYVFFFGIDNVIWIYMLTVAKVSPVVADMRVFFSSCYAFLSPYFIISSNKKVKA
KIVCAAGQDQPSADNQESSDK

>Pol_ORA1

MDLCVTIKGVSFLLQTGMGILGNAVVLLAYSHIIYTEPKLLPVDMILCHLAFANLMLL
LTRCVPQTMTVFGLRDLLNDGSCKVVIYAYRIGRALSVCITCMLSVFQAVTIAPAGPRL
SRLKPALPSLVLPTFAGLWLLNMAICIAAPFFSMAPRNGTVPAFTLNLGFCHVDFRDNL
SYVINGVAVSLRDFAFVALMLGSSGYILVLLHRHSHQVRGIRRSQGGGAETRAAKTVI
TLVVLYVVFFGIDNVIWIYMLTVAKVSPVVADMRVFFSSCYASLSPYFIMSSNKKVKA
KIMCASEQEQPSADTQESNDK

>Mar_ORA1

MDLCVTIKGVSFLLQTGMGILGNTVVLLAYVSIIFTEPKLLPVDMILCHLAFANLLLVL
TRCVPQTMTVFGLRDLLNDPGCKVVIYAYRISRALSVCITCMLSVFQAVTIAPAGPHLS
KLKPVLPSLVLPTFAGLWVLNMAICIAAPFFSMAPRNGTVPAFTLNLGFCHVDFRDNL
SYVINGVAVSVRDFAFVALMVGSSGYILLLLHRHSRQMRQLRRSQGGGAETKAAKTV
ITLVILYVVFFGIDNVIWIYMLTVPKVSPVVADMRVFFSSCYASLSPYFIISSNKKVKAKI
VCAAEQDQPSADTQESNDK

>Cau_ORA1

MDLCVTIKGVSFLLQTGMGILGNTVVLLAYAHIICTEPKILPVDMILCHLAFANLMLLL
TRCVPQTMTVFGLKDLLNDPGCKVVIYAYRIGRALSVCITCMLSVFQAVTIAPAGPRL
SRLKPALPSLVLPTFAGLWLLNMAICIAAPFFSMAPRNGTVPAFTLNLGFCHVDFRDSL
SYVINGVAVSGRDFAFVALMVGSSGYILLLLHRHSRQVRGIRRSHGGGAETRAAKTV
VTLVILYVVFFGIDNVIWIYMLTVAKVSPVVADMRVFFSSCYAALSPYFIISSNKKVKA
KIVCAAEQDQLSADGPESNEK

>Ame_ORA1

MDLCITIKGVSFLLQTGLGIMGNMLVLAAYGHIALVEPRLQPVDQIMAHLAFANLML
LLTRGVPQTMTVFGLRHLLNDSGCKVVIYTYRITRALSVCFTCMLSVFQALTIAPAGG
PRLARLKARLPQLVAPTFAGLWLLNMAVCIAAPFFSVAPRNGTVPPFTLNLGFCHVDF
RDNLSYVINGAAVSARDFSFVGLMLGSSGYILVLLHKHSRQVRAIRRSQGGSMEMRA
AKTVVMLVVLYAVFFGIDNVIWIYMLTVAQVPAVVADMRVFFSSCYATLSPFLMISSNK
KLKERMVCAAGGEQKQDAAEDTDKTNIK

>Lin_ORA1

MQFKEFVHGMPYLSFTRVGVSGNIVVIWAFLLALYQDNQFLPANAIVLHLACSNLLV
VGMHCLLETLASFRQVSVXGDVCCKAVIFVYRTSYSLSIWLTFVLSAHQCLSIAPPGS
RWASIRALAASYLVFVFFLLSVINTCMSSTDMXFFLGTQNDSNLMNHGNNVQFCYVR
FPSKLIKDANGATQVGRNMTPMALMILSSVIILVFLYRHSKPSS

>Dre_ORA1

MDLCVTIKGVSFLLQAGLGILANALVLLAYAHIRLAEARLQPVDAILCHLALVDLLLL
LTRGVPQTMTVFGMRNLLDDTGCKVVIYTYRIARALSVCITCMLSVFQAVTVAPAAG
PLLSGVKARLPQLLAPTFAALWFINMAVCIAAPFFSVAPRNGTVPPFTLNLGFCHVDFH
DNLSYVLNGVAVSVRDFAFVGAMLASSGFILLLLHRHRRQVRAVRRSQGSTMETRAA
RTVLMLVILYSVFFGIDNVIWIYMLTVAQVPPVVAHMRVFFSSCYASLSPFLIISSNRKL
KARMVCATSEQERQAEDGKNSSGKN

>Gmo_ORA1

MDLCVTIKGVSFLLQTGLGVLGNALVLLAYVHIAHGADHKLLPTDLILCHLAFSNLVL
LLTRCVPQTMTVFGLHDLLDDAGCKVVIYLYRITRALSVCLTCMLSVFQAATLAPDAP
RLKAALPALVLPSFAGLWLLNMAVCIAAPFFSIAPRNGTVPAFTLNLGFCHVDFRDYLS
YVINGVAVSVRDFAFVLLMLLSSGYILLILHRHSRQVRSMRRGAAQGVETRAAKTVV
TLVVLYAVFFGIDNVIWIYMLTVAKVSPVVADMRVFFSSSYAFLSPYFIISSNKRIKGKL
VCAVEQQQSPVETQTSNEK

>Gac_ORA1

MDLCVTIKGVSFLLQTGMGILGNTVVLLAYAQLIYAEPKLLPVDMILCHLAFANLMLL
LTRCVPQTMSVFGLRDLLGDPGCKVVIYAYRIGRALSVCVTCMLSVFQAVTLAPAGPR
LSRLKPALPSLVLPTSAGLWLLNMAVCVAAPLFSMAPRNGTAPAFTLNLGFCHVDFRD
NLSYVINGVAVSVRDFAFVALMLGSSGYILLLLHRHSRRVRGIRRSQGGGAETRAAKT
VITLVVLYAVXFGIDNAIWIYMLTVAKVSPVVADMRVFFSSCYASLSPYFIISSNKKVKA
KILCAAEQDQPSVDNQETSDK

>Loc_ORA1

MDLCITIKGVSFLLQTGLGILGNLLVLLAYAHIACSDGRVQPVDKILCHLAFANLLLLL
TRCVPQTMTVFGLKDLLNDSGCKAVIYAYRIARALSVCITSMLSVFQSIIIAPASSRWV
GLKVRVSQLLFPSFAALWLINMAVCIAAPFFSIAPRNGTVPEFTLNLGFCHVDFRDSLS
YIINGVAVSGRDFIFVGLMVCSSGYILLLLHRHGKQVRQIRSPDQHSSRNAAETRAAK
TVVTLVTLYVVFFGIDNIIWIYMLTVAQVPPVIADMRVFFSSCYASLSPFLMITSNKKIK
NKLSCAAANPEQMSLNTEDSTRI

>Ola_ORA1

MDLCVTIKGVSFLLQTGLGILGNSVVLLVYSHIMCTGPKLLPVDMILCHLAFANLILLL
TRCVPQTMTVFGLKDLLNDPGCKVVIYAYRIGRALSVCITCMLSVFQAVTIAPAGPFLS
RLKLALSSLVFPTFVGLWLLNMAVCIAAPFFSMAPRNGTVLPFTLNLGFCHVDFRDNL
SYVINGVAVSVRDFAFVALMVGSSGYILLLLHRHSHQVRKIRRSHSSGAETRAAKTVL
ILVILYVVFFGIDNVIWIYMLTVSNVSPVVADMRVFFSSCYASLSPYFIISSNKKVKRKIV
CAAEQDQPSVETQESNDK

>Can_ORA1

MDLCVTIKGVSFLLQTGMGILGNAVVLLAYANIIYTEPKLLPVDMILCHLTFVNLILLL
TRCIPQTMTVFGLTDLLNDSGCKVVIYAYRISRALSVCLTCILSVFQAVTIAPAGPRLSR
LKTALPSLVLTTFVGLWLLNMAICIAAPLFSTAPRNGTVPAFTLNLGFCHVDFRDNLSY
VINGVALTIRDFAFVALMVGSSGYILLLLHRHSLLVRGMRRSHGGGAETRAAKTVITL
VVLYVVFFGIDNAIWIYMLTVTKVSPVVADMRVFFSSCYAFLSPYFIISSNKKVKAKIA
CAAVQDQPSVDNQESNDK

>Cge_ORA1

MDLCVTIKGVSFLLQTGLGILGNTVVLMAYAHIVYTEHRLLPVDMILCHLAFANLML
LLTRCVPQTMTVFGLRELLDDAGCKVVIYSYRIGRALSVCITCMLSVFQAVTIAPAGP
RLSRLKATRSSLVLPTFAALWLLNMAICIAAPFFSIAPRNGTVPAFTLNLGFCHVDFRD
NLSYVINGVAVSVRDFAFVGLMLGSSGYILLLLHRHSRQVRGIRRSQGGGAETRAAKT
VVTLVVLYVVFFGIDNVIWIYMLTVAKVSPVVADMRVFFSSCYAALSPYFIISSNKKVK
AKIVCAADQDQPSVDTKQSNDK

>Xma_ORA1

MDLCVTIKGVSFLLQTGLGILGNAVVLLAYASIICTEPKLLPVDMILCHLAFANLMLLL
TRCVPQTMTVFGLKDLLNDPGCKAVIYAYRIGRALSVCITCMLSVFQAMTIAPAGPKL
SKLKPMLPSLVLPTFAALWLLNMAICIAAPFFSMAPRNGTIPAFTLNLGFCHVDFRDN
MSYVINGVAVSGRDFAFVALMLGSSGYILLLLHRHSEQVKGIRRSHGSRSETRAAKIV
VTLVVLYVVFFGIDNVIWIYMLTVSKVSPVVADMRVFFSSCYASLSPYFIISSNKKVKR
KIVCVAEHDQPLVDTQESNEK

>Ssa_ORA1

MLDLCVTIKGMSFLLQTGLGFLGNTLVLLAYTQVVCSECRLQPVDIILCQLAFVDLILI
LTRCIPQTMTVFGLRDLLNDPGCKVVVYSYRIARALSVCITCMLSVFQAVTIAPAGGP
CLSRLKAQLPSLIVPTIAGLWLFNMAVCLAAPLFSIAPRNGTVPAFTLNLGFCHVDFRD
RLSYKINGVVVSTRDFAFVGLMLWSSGYILLLLHRHSHQVRSIRRSSQGGGAETRAA
KTVITLVVLYAVFFGIDNIIWVYMLTVDKVSPVVNDMRVFFSCCYACLSPFFIISSNKKV
KSKLVCVAADQEQPSVNTQDSNDKM

>Oni_ORA1

MDLCVTIKGVSFLLQTGMGILGNTVVLLAYTHIVCTGPKLLPVDMILCHLAFANLLLL
LTRCVPQTMTVFGLKDLLNDPGCKVVIYAYRIGRALSVCITCMLSVFQAVTITPTGPYL
SRLKPSLPSLVLPTFAGLWLFNMAICIAAPLFSMAPRNGTVPAFTLNLGFCHVDFRDNL
SYVINGVAVSGRDFAFVALMVGSSCYILLLLHRHSHQVKGIRRSQGGGAETRAAKTV
LTLVVLYVVFFGIDNVIWIYMLTVAKVSPVVADMRVFFSSCYASLSPYFIISSNKKVKA
KIVCAAEHEQPSADTQDSNDK

>Oni_ORA2

MASEVFVRGMLYLSLTIVGIPGNATVIVAFLLLLYQEKRLLAADAILLHLACVNLLVV
VVRALTETLASFRLADIFGDTGCKSVIFIYRTTRALSIWLTFLLSTYQCLSIAPPGSSWA
SVRALLGHYLAFVFLFLWVLNTCMTTAAILFSFSTKNETSPIDNGINVQFCYVNFPSM
LSRDANGAVQVGRDVVPMALMTLASLIILVFLYKHSQQVKGLRSSGGGGAGNGGAE
QRAAKAVVALVTLYVVFYGVDNVLWVYTLTVKKTMSSSLISDLRIFFGSLYAALSPLVI
IASNRKVNSRLGCVAHEKSAVEKTKNLSSM

>Ssa_ORA2

MQSEEVVRGMLYLSLTVVGVPGNTAVIVAFLLALYQEHQLLPADAIVLHLACANLLV
VGVRCLLETLATFRLVNIFGDTGCQGVIFVYRTSRSLSIWLTFVLSAYQCLSIATPGSRW
ASIRVLVARYLAVIFLTLWVINTSMSSAAIAFSLGSRNDSVNMQHSINVQFCYVRFPTM
QSKQVNGAVQVGRDVVPMGMMTLASLVILVFLYRYSQQVKGLRSSSGASGGAERRA
AKAVVVLVTLYVVLYGVDNGLWVYTLTVRKTMSSSLISDLRIFFSSLYAALSPLVIIATN
RKVNSRLRCVVQERPVQDKATTLSTV

>Xma_ORA2

MPSNEDIRGMLFLSLTVVGVPGNMAVIVAFLLLILQESCLLAADAIVLHLSCTNLVVVL
VRCLMETLASFHLANVFGDIGCKGVIFIYRTSRALSIWLTFLLSAYQCLSIAPPGSKWA
SLRTLVAQSLPIVFVFLWVLHSSLSAGAILFSVSSKNVTAVATSAVNVEFCYVNFPSDIL
KKVYGAIQVSRDVVPMALMTLTSLIILVLLYKHSQHLKGLRGAGHAGSGTGGSKQRA
AKVVVVLVTIYVVLYGVDNCLWVYTLTLRHTMSSSLISDLRVFFASLYAALSPLVIIVS

NRKVNSRLRCVAQEKPLLGKTAHLHSI

>Cge_ORA2

MPSEEFVRGMLYLSLTVVGVPGNTAVILAFLLALYQENRLLPADAIILHLACVNLLVV
GVRCLLETLASFSLANVFGDTGCKAVIFVYRTSRSLSIWLTFVLSAYQCLSIAPPGSRW
ASVRVLGAHYLGLVFLVLWVINTCMSSAAILFSLGTQNDSSLMSHGINVQFCYVRFPS
KLSKQANGAAQVGRDVVPMALMTLASLIILVFLYKHSQQVKGLRSSSSSSGGSGGSE
QRAAKAVVALVTLYVVLYGVDNGLWVYTLTVRKTMGSSMISDLRIFFSSLYAALSPLV
IIASNRKVNGRLRCVVQEKPIQ

>Can_ORA2

MPSEVLVRGALYLSLTVVGVPGNAAVILAFLLLLYQESRLLPSDVIVLHLACVNMLVV
VVRCLLETLASFRLAVIFGDVGCKAVIFVYRTCRSLSIWLTFALSAYQSLSIVPPGSRWA
SFRHTVAQHLGFVFLFLWLLNTCMSSAAILFSFGAGSNSSAANHGINVQFCYVKFPSK
LSIEANGAVQVGRDVVPMALMTLASLIILVFLYRHSRQVKGLRSSGGGAEQRAAKAV
VALVTLYVVLYGVDNGLWVYTLTARKAMASSLISDLRIFFSSLYATLSPVVIIASNRKV
NGVLRCDVRRKPVQERDTCLSTM

>Ola_ORA2

MASEVFVRGMLFLFLTVVGIPGNATVIVAFLLLLYQEKRLLAADSILLHLACVNLLVV
VVRALTETLASFRLADIFGDTGCKSVIFIYRATRGLSIWLTFLLSTYQCLSIAPPGSSWA
SVRALLGHYLAFVFLFLWVLNACMTTAAILFSFSTKNETSPIDNGINVQFCYLNFPSKL
SRDANGAIQVGRDVVPMALMTLASLIILVFLYKHSQQVKGLRSSGGGGAGNSGAEQR
AAKAVVALVTLYVVFYGADNGLWVYTLTVKKTMSSSLISDLRLFFGSLYAALSPLVIIA
SNRKVNSRLGCVAHEKSAVEKIKNLSSM

>Sch_ORA2

MASEEFVRGMLYLSLTVVGIPGNIAVILAFLLLLYQENRLLPADAIVLHLACVNLLVVV
VRCLLETLASFRLASIFGDLGCKAVIFGYRTFRSLSIWLTFVLSAYQCLSIAPPGSRWAS
VRTLVAHYLWFLFLVLWLINTCMSSAAILFSFGTKNNSSLINHGINVEFCYVYFPSKLS
KEANGAAQVGRDVVPMALMTLASLIILVFLYKHSQQVKGLRSSSSGGGGGSGGAEQ
RAAKAVVALVTLYVVLYGVDNGLWVYTLTVRTTMKSSLISDLRIFFSSLYAALSPVVII
ASNRKVNGRLRCVVQEKPVQEKATSLSTM

>Ame_ORA2

MDLYFLTRGLLYLFLPVFGVPGNCAVIWAFLLALRQEGTLLPADAIVLHLACANLLVV
SCRCVFEVFANFQVFNGFNDPGCKGIYFIYRTFRGLSIWLTFTLSSYQCLSIAPPGSHW
ATLRSLFGRYLWLIFLLLWIINTSASAPTLVFAVAARNDSKLLENSINIQFCFINFPSVFA
KDANGALQVVRDVIPMSLMTTASFIILVFLYRHSRQVSNLRSGTGTGGGGASAERRA

AISVVVLVTFYVLMYGVDNGLWVYTLTVKQTLSSALISDLRIFFSMLFAAISPIIIITTN
MKVKKQLL

>Dre_ORA2

MIAEAVIRGLLFLSLVLVGVPGNTAVICGFILLVRREGRLSPADAIVLHLCSANLVVVSV
RCLLEVLATFRIHNVFDDAGCRAVIFLHRTARSLSIWLTFLLTALQCLSVAPPGSRRAAA
RALLARSLPAIFLALWLINTSMSVASLLYSIGARNDSRLLQNAINVEFCFLSFPSRLARD
ANGAAQVARDVVPMVLMAAGSLVLLVYLVRQRRRVQGLRGTAGGAAERRAAVTVV
TLVSLYLLVFGLDNGLWVYTLTVSHTLSSALITDLRLFFTSLYTAVSPLLILVSNTRLRC
GKQPETMH

>Gmo_ORA2

MPSEELIRAMLYLTLTVVGVPGNLAVIWAFLLALHQERRLLPADTILLHLASVNLLVV
GVRCLLETLASFRLASVFGDTGCKSVIFVYRTARSLSIWLTFVLSAYQCLSIAPPGSRW
AAARALAARYMAAIFLALWLGNTCMSSAAVLFSVGAGNGNGSSSLGSNGINVQFCV
VRFPTRLSKDANGAVQVARDVVPMGLMATASLVILVFLYRHSRQVKGLRSGGGGGR
DGAERRAAKAVVALVTLYVGLYGVDNGLWVHTLTVRRTMGSSLVSDLRIFFSSLYAA
LSPAVIIATNRKVQRRLRCGRGEKHRGESATEATAVSTM

>Gac_ORA2

MPSEMFVRGMLYLSLTVLGVPGNATVILAFLLLLYQERRLLPSDAIVLHLAFVNLLVV
AARCLPETLASFRLSGIFGDVGCKAVIFVYRTSRSLSIWLTFVLSAYQCLSIAPPGSRWA
HLRVLLAQYLGLVFLILWLLNTCMSSAGILFSFGTKNVTNLTNFDINVQFCYVNFPSKL
SIQANGASQVGRDVVPMALMTLDSLIILVFLYKHSQQAKDLRGSRGGGAERRAAKV
VVALVTLYVVLYGVDNGLWVYTLTSRKAMESSLISDLRVFFASLYAALSPAVVIASNR
KVNSRLRCDVKRKPVEEKDTCLSTV

>Loc_ORA2

MDPQVVIRGMLYLFLVVVGVPGNLAVIWAFCHIMRSERKLMPADAIVLHLAAVNLLV
AAVRCSFEALAAFGVLYVFNNTGCKTIIFIYRTSRSLSIWLTFVLSTFQCISIVPPGSRGY
SIKSHAPRYLGGVFVFLWILNSWLSSAALAFAVSSGDNSTRTQYGINIEFCIVNFPSSTW
KNAVGAVQVARDAVPIFLMVAASLFILLFLYRHSQQVKGLRSAKRTQKESAESRAAKT
VVTLVTLYVLFYGIDNGLWVYTLTVTQTLSTSLISDLRIFFASLYAAVSPLVIIASNKKV
KSQLGCMKTEKGPVSVDTVLSTV

>Tni_ORA2

MIVVTIILRDLLSISPGMQSTEFVRGILYLSLAVVGAPGNTCVILAYFILLYQEKRLLPAD
VIILHLACANLLVVVARCFLEFLASFRLALIFGDVGCKSVIFVYRTSRSLSIWLTFILSAY
QCLCIAPLGSQLATLRMIVAKYLFYVFFFLWLLTTTMSTAAILFSFSTQNGTNLVNNSIN

VQFCYVQFPSKLSKDANGAAQVGRDVVPMTLMTLASLIILAFLYKNSQQVKGLRSSS
SDRAEKRAAKAVVTLVSLYVLLYGVDNGLWVYTLTVREAMASSLISEMRIFFSSMYA
ALSPIVIIVSNRKVNNILRCAGQEKHVQEKTT

>Cau_ORA2

MPSEELVRGMLYLSLTVVGVPGNTAVIVAFLILFYQENRLLAADAIVLHLACANLLVV
GVRCLLETLASFHVANVFDDTGCKAVIFVYRTSRSLSIWLTFLLSAYQCLSIAPPGSHW
ASVRTFVAHYLAVLFLFLWIVNTCMSSAAIMFSFSTQNASSPMNNGINVQFCYVLFPS
KLSKEANGAAQVGRDVVPMILMTLASLMILVFLYKHSQQVKGLRSSSGGGGSGGAE
QRAAKAVVALVTLYVVLYGVDNGLWVYTLTVRQTMSSSLISDLRIFFSSLYAALSPLVI
IASNRKVNSRLRCVAQEKPVLEKATSLSTM

>Tru_ORA2

MKDYGGIILRDLFSISTGMQSVEFVRGILYLSLTVVGAPGNICLILAYLILLHQENRLLP
ADVIILHLSCVNLLVVVARCLLEFLASFHLAIIFGDVGCKSVIFVYRTSRSLSIWLTFILS
AYQCLCIAPPGSQWTTLRIVFASYLFYVFFFLWLLTTSMSSAAVLFSFGTQNDTNLINH
SVNVQFCFVHFPSKMSRDANGAAQVGRDVVPMALMTLASLIILAFLYKNSQQVKGL
RSRDGGSGRAERRAAKAVVTLVTLYVLLYGVDNGLWVYTLTVREAMRSSLISDLRVF
FSSLYAALSPIVIIISNRKVNSILRCAEQQKHVQ

>Cau_ORA3

MGHDENTELVGMGLRVSVSPVQTTFYIFLVMLGVLGNATVIVVIGKSVLMDRGGGR
NSDIIIMNMALSNLLVSVLRNTLLVISDIGLEVLYSSKEWCQFLMGIWVWLRSVNVWS
TLFLSAFHLQTLRRVAPTAGVLHGSRGVPMTLLLSLGLIWLLNFIYAIPAHIFSTNGNV
NTTQTLMLVSSTTRPLLGCVWNFPSSYSGLAYATTSMVIHETIPIVLMALTNLSSLYTLY
THGRVRSSVKDGPVIKRVPAERRAAKVRNW

>Loc_ORA3

MGEQTNKVVTVLLKAAIPAQNALYGLLVMLGIVGNGLVMGVVGRGLVKEGLARQH
SDIILLNLVLSNLLVSLVRNIPLLLADVGLQLFTSPGCCQFLMFMWVWLRSVNVWMT
MCLSAFHFLTLCRLGPVVPAGPHGPRASLQRLLLVLALIWSLNLLYSFPGFFFSTQGGR
NSTEELMLVSSTTRPLLGCVWSFPSRRGGLAYATTSLVLHELLPILLMVATNLGTLHTL
ARHGRSQRAGETTLTRRIPAERRAAKVVLVLIMLFIISWGASVLSVNYYNYNRGPSTE
FLLVMARFTNSLFIAFSPLVLLAGHSRLKAIFRVIADHVHSFCFSQRCLKKSC

>Ssa_ORA3a

METPMPELKELEPVGVGLRVTTSPTQTTFYIILVLLGIVGNTTVIGVMLDSVFKDPSGV
RNSDIILMNMALSNLLVSVLRNVLLVISDLGLELNTSRDGCHVLMGVWVWLRSVNV
WSTLFLSAFHFQTLRRVAPPPGTVHGPRRPPKTLLISLGLIWFLNLIYAVPAHIYSTKGN

KNSTEILPIILMAITNLGSLYTLYTHGRTHNPAHMTQDAPVIKRIPAERRAAKVILALIV
LFIGSWGTSIISINYFNYNRGLSAGFLLVIARFANTIFIAISPIVLALGHRRLRAVIKYFLT
H

>Ssa_ORA3b

METPMPELKELEPVGVGLGVNRYPFQNALYIIFVLLGIVGNATVVGVISESVFKDPSG
GRNSDIILINMALSNLLLSLLRNILLVISDLGLELNTSRDGCHVLMGVWVWLRSVNV
WSTLFLSAFHFQTLRRVAPPSGTVHGPRRPPKTLLISLGLIWFLNLIYAVPAHIYSTKGN
KNSTETLMLVSSTTRPLLGCVWNFPSSYDTLAYTTTSMVIHEILPVILMAITNLGSLYT
LYTHGRTRNPAHMTQDAPVIKRIPAERRAAKVILALTILFIVSWGTSIISINYLNYYKGS
SATFLPVIARFANSIFIAISPIVLALGHRRLRAVIKSFLTH

>Gac_ORA3

MAEHIGEDGESTLIGMGLRVSVSPVQTAFYIMLVTLGILGNFTVVGVIGKSIATDHVGG
RNSDIIIINMALSSLLVSVMRNIPLVISDIGLELYSSKEWCQVLMGLWVWLRSVNVWST
LFLSAFHLQTLRRVAPTAVSRNGPRGLPKTLLLSLTLIWLLNLVYSIPAHIFSTSGDVNST
ETLMLVSSTTRPLLGCVWNFPSSYSGLAYATTSMVIHETIPIILMAITNLGSLYTLYTHS
RVRSTDAPVIKRVPAERRAAKVILTLIMLFIVSWGTSIISVNYFNYNRGSSAEYLLIIARF
ANIIFIAMSPIVLTFGHRRLRSFVKSTLSH

>Gmo_ORA3

MSEAEAELVGMGLRTDASPVQTTFYILLVLFGIVGNTTVIGVIGHSVLMNPGVGRNSD
IIIINMAVSNLMVSVLRNALLVISDIGIALYSSKECCQFLMGVWVWLRSVNVWSTLLLS
AFHFHTLRRVAPPLGNLHGPRGLPKLLLLGLGLIWVLNFLYSIPAHVFSINGNQNSTET
LMLVSSTTRPLLGCIWNFPLKNGLLYATISMVIHETLPIVLMAFTNAGSLYSLYAHSKM
RSMVNDVHVIKKVPAERRAAKVILALIMLFIASWGTSIISVNYFNYNRGQSAEFLLVIA
RFANIFFIAMSPIILSIGHRRLRSFFTSLV

>Lin_ORA3

MADSDDAELVGMGLRVSVSPVQTAFYVLLVLIGVVGNATVVGVIGKSILKDHGGGR
NSDIIIVNMALSNLLVSVMRNTLLVISDVGLELYSSQEWCQFLMGIWVWLRSVNVWS
TLFLSAFHLQTLRRVAPPVGHPQGTRGPPKSLLLTLGLIWLANFIYSIPAHIFSKSGNQN
TTETLMLVSSTTRPLLGCVWNFPSSYSGLAYATTSMVIHETIPIILMTITNLGSLYTLYAH
SKLRSLLQDAPVIKRVPAERRAAKVILSLIMLFIASWGTSIISVNYFNYNRGSSADFLLL
IARYANIIFIASSPIVLAVGHRRLRSFIKSVIAH

>Dre_ORA3a

MAPQKKPVNISQRITSSPFYIMLYVVLVLLGNAGNTTVIAVVGQSLLQETGTVRSSDVI
LVNMAFSNLMVSLLRNTVLMVSDLGVEIFLSRDMCQFMMGLWVWVRSANVWSTFF

LSAFHFQTLRRVAPPVINLHGPRGPPLSLILGFCLIWSLNLIYSIPAFIFSKNGNENSTETL
MLVSSTTRPLLGCIWDFPSAYSGLAFATSSMILHESIPICLMNITNLGSLCTLYAHGHKR
TVASQGEDAPVVSRIPAERRAAKVILALNILFISSWGTNVISVNYFNYNRGQSTEFLLII
ARFVNMSFIAFSPIILAVGHRKLRAFIKSVLSHMI

>Dr_ORA3b

MATTAKPLTVSQRALSSPLYIAFYVILVLLGNLGNSLVIGVVGEGLLREPGVARSSDIIL
VNMALSNLMVSLTRNSLLVISDMGVQVFLNRNWCRFMMGIWVWVRSANVWSTFFL
SAFHFQTLRRVAPPVSNVHGHPGPPRSLIFGLCLIWSLNLIYSIPAFIFSKNGDANSTETL
MLVSSTTRPLLGCIWNFPSAYSGLAFATSSMILHESIPICLMSITNMGSLLALYAHGEAR
RAAKKSSDAPVVSRIPAERRAAKVILALNILFILSWGTSVISVNYFNYNRGSSTDWLLI
AARIGNITFIALSPIVLAVGHRRLRAFLASILTHSIALCRHLWTYKLQK

>Sch_ORA3

MAAMNGSTEENGNTKLPGMGLRVSVFPVQIAFYIGLVILSILGNATVIGVIGKSLIMDR
GGGRNSDIIIINMAVSNLLVSVMRNILLIISDIGLQLYLSKDWCQFFMGVWVWLRAVN
VWSTLFLSAFHFQTLRRVTPVIGNLHKSQGASTTLLLSLSLIWLLNFIYAIPAHVFSTNG
NKNSTETLMLVSVTTRPLMGCVWNFPSSYSGLVYTTSSMVIHEVIPIILMTFTNLGSLY
TLYTHSRMRSSVQDALFTKRIPAERRAAKVILAIIILFIGSWGTSIITVNYFNYNRGSSTE
FVLVIARFSNSIFIAVSPAILAVGHRRLRSFVKSMLTH

>Ame_ORA3

MWVNGTVTIIKATGGQLSSVPMALYMILVLLGIFGNAIVISVVGESILREPGGGRNSD
MILVNMAFSNLMVSMTRNMLLVISDTGLEVLPGKDWCQILMGVWVWLRSVNVWST
FFLSAFHFHTLRRTAPPITSLSGPRGLPRGILTGFGLIWSSNLLYSVPAFIYSTSGGKNAT
ETLMLVSSTTRPLLGCLWDFPSVYSGLAFATTSMVIHEIIPIVLMSVTNLGSLLTLYAHG
SKLHATNKSQQDPTMNRVPAERRAAKVILALILLFIVSWGASVISVNYFNYNRGASST
YLLVLARFFNSLFIALSPLILAVGHRRLRQFFKSIISH

>Ola_ORA3a

MSALQAVFYAILVVLGVLGNTTVIVVVGKSVIQDRRVAHNSNIIIINMAASNLMVSVM
RNILLVMSDFGIQLFLSRERCQFLMGVWVWLRSVNVWSTFYLSVFHLQTLRRVAPSV
GNLQASRGVPKTLLLNLLSIWILNLLYSIPAHIFSTNGNANSTETLMLISSTTRPLLGCV
WNFPSSYSGLAYATTSMVIHETLPIVLMTVTNLSSLYTLHTYGRTRKSVQDAPVVKRV
PAEKRAAKVILILVLLFTVSWGTSVISVNYFNYNRGTSSEFLLVIARFAQILFIALSPAVL
AVGHRGLRSCIKSSLTY

>Ola_ORA3b

MSAVSRMKTQLDLRPADEAAAKIKDFVGPEGRDENPKHVGIGLQVPVSDVQIVCHVI

MVVLAILGNATVIVVIGKSVIQDRRVAHNSNIIIINMAVSNLMVSIMRNILLIVSDFGIQL
FLSRERCQFLMGVWVWLRSVNVWSTFYLSVFHLQTLRRVAPSVGNLQASRGVPKTL
LLNLLSIWLLNLLYSIPAHIFSTNGNANSTETLMLISTTTRPLLGCVWNFPSSHSGLAYA
TTSMVIHEALPILLMTATNLSSLYMLHTYSRTRTRTSIQHAPVIRGVPAERRAARVILIL
VLLFVVSWGTSVISVNYFNYNRGTSSEFLLVIARFAQILFIALSPAVLAVGHRGLRSCIK
SSLTY

>Can_ORA3

MEGNEEAMLVGMGLRVSVSPVQTTFYLLLVILGILGNSTVVGVIGKSVLTDHAGGHN
SDIIIINMALSNLLVSVMRNIPLVISDIGLEVQLYSSKEWCQVLMGVWVWLRSVNVWS
TLFLSAFHLQTLRRVTPMSSTNGHRGLPKTLLLSLALIWLLNFIYSIPAHIFSTSGDVNS
TETLMLVSSTTRPLLGCVWNFPSSYSGLAYATTSMVIHETIPIILMAFTNLGSLYTLYTH
SRVRSSAQDAPVIKRVPAERRAAKVRNTRVAGGGFLVKSSL

>Cge_ORA3

MEENGETELVGMGLRVSVSPAQTAFYIFLVLLGILGNATVVGVIGESVCTDPSGGRNS
DIIIINMALSNLLVSVLRNTLLVISDLGLEVLYSSKEWCQVLMGVWVWLRSVNVWST
LFLSAFHLQTLRRVAPPIANLHGPRGPPKSLLLGLGLIWLVNFIYSIPAHMFSTSGNANS
TETLMLVSSTTRPLLGCVWNFPTSYSGLAYATTSMVIHETIPIILMAFTNLGSLYTLYAH
SRMRSSVQDAPVIKRVPAERRAAKVILALIMLFIASWGTSIISVNYFNYSRGSSAEFLLV
IARFANIIFIAMSPVVLTIGHRRLRSVIKSLLSH

>Pol_ORA3

MSSEVFVRGMLYLSLTVIGVPGNTAVILAFLLLLHQENRLLPADAIVLHLACMNLLVV
GVRCLLETLVSFELADVFSDVGCKAVIFIYRTSRSLSIWLTFVLSAYQCLSIAPPGSRWA
SARALVARYLGCVFFFLWLLNTSMSSAAILFSFGKKSNSTMINHSINVQFCYVQFASKL
SKEANGAVQVGRDVVPMALMTLASLIILVFLYKHSQQVKGLRSSRGGGGGSGGAER
RATKAVVTLVTLYVVLYGVDNGLWVYTLTVRQTMKSSLISDLRIFFSSLYAALSPVVII
ASNRKVNGQLRCVAQEKPIQEKATSLSTM

>Tni_ORA3

MAANTEDDTEVVGMGLRVSVSPVQTASYIFLVLLGILGNTTVVGVIGKSILMDRGGG
RNSDIIIVNMALSNLLVSLMRNTLLILSDLGLEMYSSKEWCRFLMGVWVWLRSVNV
WSTLFLSAFHFQTLRRVAPVAGPVQGARGAPKILLINLFLIWFINLIYSIPAHIFSTSGNI
NSTETLMLVSSTTRPLLGCIWNFPSRYSGLAYATTSMVLHETVPIVLMALTNLGSLYTL
YTHNGMRSSVQEVPIKKRVPAERRAAKVILALIMLFIISWGTSVISVNYFNYNQGSSAE
FLLVIARFANIIFIALSPVVLAVGHRGLRSFFKSLLAH

>Mar_ORA3

MGRRIIASPVQISFYFILLIMGILGNTTVVRVLGKSIFVDPTGTRNSDIIIFNMALSNLVV
CLMRNTVLAISDLGLEVCSLSKLSCQFLMGVWVWLRSVNVWSTFFLSAFHLLTLRRV
TPMFGNLQRGMSKPLLLSLGIIWFFNYICAIPAHIFSTKGGVNTTETLMLVSSTTRPVLS
CVWNFSSTYSGLAYATTSMVIHEILPILLMIFTNICSIYTLYKHASRRSLVQDASVIKRIP
AERRAAKVQVILMLITLFIASWGTSVISVTYFNYKRSSSSEFFLIITRFSNIIFIALSPAAL
AFGHRQLRSFIKSFLTY

>Xma_ORA3

MEQETTTKLVGMGFRTAVSPAESDFYIILVALGIVGNSIVIGVIGKNVMMDRGPGHNSD
IIIVNLAVSNLMVSIMRNLLLIISDLGFKLYSSKGWCQFLMGVWVWLRSVNVWSTFFL
SAFHLHTLKRVTPTIGDLQGPRSTYRTLLLSLAIIWILNFLYSIPAHIFSTSGNENTTETL
MLVSSTTRPLLGCVWNFPSNYSGLAYATTSMMVHEIFPIILMVVTNMMSLYILNTYGR
SRGSVQDVPVLKRVPAEKRAAKVILALVMLFIVSWGTSIISINYFNYNRGSSAEFLLVV
ARFGNSIFITMSPVVLAIGHRRLRSCMKSSVSD

>Tru_ORA3

MAVNSMDEDDMELVGMGLRVSVSPFQTAFYIFLVLMGILGNATVVGVIGKSIIMDRG
GGRNSDIIIVNMALSNLLVSLMRNMLLILSDIGLEMYSSKEWCQFLMGVWVWLRSVN
VWSTLFLSAFHLQTLRRVAPMAVNVTGSRGAPKILLMNMFLIWFINLLYSIPAHVFSTS
GNINSTETLMLVSSTTRPLLGCIWNFPTRFSGLAYATTSMVLHETVPIVLMALTNLGSL
YTLYTHNGMQSSVQDAPVIKRVPAERRAAKVILALIMLFIASWGTSIISVNYFNYNQG
SSAEFLLVIARFANIIFIAMSPAVLAVGHRGLRTFFKSLLSH

>Oni_ORA3

MSSLSDQRKEPDLAGMGMRIFVSPAQTAFYIILVIMGILGNTTVILVIGKSIILEHNWGR
NSDIIIVNMAMSNLLVSLLRNTLLIISEIGLQIYTTKGFCQLLMGMSVWLRSVNAWSTL
FLSAFHLQTLKRVAPGATNGPRGVPKTLLVCLGLIWIGNLIYSIPAHIFSSNGNKNATET
LMLVSSTTRPLLGCVWNFPSTSGLAYATTSLVIHEMIPIILMAVTNLTSLYTLYTHGRNP
RKDATVLKRVPAEKRAAKVILTLIILFILSWGTSVISVNYFNYNRGSSADYLLVIARFAN
IIFIALSPVVLAVGHRQLRSCIRSTLVR

>Oni_ORA4

MSEVFTVEAILFGLLVFSGILGNILVIYVVFQSVTKTPPRRLPPSDLILVHLSLANLLSSL
FRTVPIFVSDLGLDLSLSSGWCRVFMLLWVWWRAVGCWVTLTLSVFQCTTLRRQNV
AFGPITVQRERRRLWVVLGVVWGANLAFSVPALVYSTHVKGNATVELMVISSTTRPL
LGCVWEFPSEEQGLVFTSTSLAVNEVLPLVLMVCTNVATLHALAKHIRAVASGGIHTE
LDKHLSSERKAARVIISLVLLFVVCWVLQVAAVTYYNHNRGHHVEGLLTVSHFSSSLF

VGFSPLVVALGHGKLRRKIISMMLG

>Tru_ORA4

MSEVLTVDAILFGLLVFSGILGNFLVIHVVLQAAFQSASRRLPLSDTILVHLSLANLLTS
LFRTVPIFVSDLGSDVSLSPGWCQVFMLLWVWWRAVGCWLTLALSIFHCTTLKRQRL
YMGPDMQRRERRRMWVILGLVWGLNLAFSIPALIYSTHVHGNATVELMVISCTTRPL
LGCIWEFPTAQQGSAFASASLALNEVLPLVLMVCTNLATLHALAKHIRAVMSSGQPG
GSHVELDKHLSTERKAAQVIMLLVSLFVVCWVLQVAAVTYYNHDGGHHAEGLLTVA
HFSASLFVGFSPMVVALGHSKLRRRISSMMLGWCQCFKGRSEDDPPNTRAAKIISFVQ
HKQ

>Xma_ORA4

MSEILTVDAILFGLFVFSGILGNILVIHVVFQSALESPSRRLPPSDTILVHLSLANLLTSLF
RTVPIFMSDLGLDVSLSPGWCRIFMLLWVWWRAVGCWVTLTLSIFHCATLRRQHVTF
GPLTLQRERRRVWVVLGLVWGANLAFSIPALLFTTHIESNATMELMVISCTTRPLLGC
VWKFPSDRQGLAFASTSLALNEILPLVVMVFTNLATLHSLAKHIRAVTSESGSHGELD
KHVSTERKAAHVIMCLVSLFVVCWVLQVAAVTYYNHDGGQHAEGLLTVAHFSASLF
VGFSPLVVALGHGKLRRKIRSMILVWTNVPLSQEAESGRKSPKTSGKQISFVAQKEVK
VKKVKDKVIPRR

>Tni_ORA4

MSEVLTVDAILFGLLVFSGILGNILVIHVVLQAAFQGTSGRLPLSDTILVHLSLANLLTS
LSRTVPIFVSDLGRDVSLSAGWCRVFMLLWVWWRAVGCWLTLVLSVFHCTTLKRQR
LYIGPNAQRRERRRLWVILGLVWGLNLAFSTPALIYSTHVHGNATVELMVISCTTRPLL
GCIWEFPTAQQGSAFASASLALNEVLPLVLMVCTNLATLHALAKHIRAVVSSQSGGSQ
GELDKHLSTERKAAHLIMLLVSLFVTCWVLQVAAVTYYNHDRGHHAEGLLTVAHFS
ASLFVGFSPLVVALGHSKLRRRISKVMLSWCRRLKGSSNTKLMQIVFFVPQKQ

>Pol_ORA4

MEGSENQNDDQELVGMGLRVAVSPVQNAFYIILVMLGILGNATVVVVIGKSVIMDHG
GGRNSDIIIINMALSNLMVSVMRNTLLIISDMGLELYSSKEWCQFLMGVWVWLRSVN
VWSTLYLSAFHFQTLRRVAPTIGNLHGARGPPTTMLLSMGLIWLLNFIYSIPAHIFSTN
GDMNTTETLMLVSSTTRPLLGCVWNFPSTYSGLAYATTSIVIHETIPIILMAFTNLGSLY
TLYTHGKMRSSVQEGPVIKRVPAERRAAKVILALIMLFIASWGTSIISVNYFNYNQGSS
ANFLLIIARFANIIFIAMSPAILAVGHRRLRSTIKSLLSH

>Mar_ORA4

MSEVLILDAFLFGLLVFSGILGNILVFQAATESPSRRLSPSDTILVHLSLANLLTSLFRTV
PIFVSDLGFDVSLSPDGCRVFMLLWVWWRAVGCWVTLTLSVFHCTTLRRQHVSFGAL

SQQRDRRRIWIVLGVVWGANLAFSLPALVYSTHVYSNTTVEMMVISCTTRPLLGCV
WEFPSKEQGSAFASTSLALNELLPLVLMVCTNLSTLHALTKHIRAVTSGGESGGVLER
QVSTERKAAHVIMALVFLFVVCWVLQVAAVTYYNHNRGQHSEGLLTVAQYSASLFG
GFSPLVVALGHGKLRKRIMSMILGWSKALKRHSKDTESPKTKEERRKQTIFVVPKGSD
VRKVEGNVIK

>Cge_ORA4

MSEVLTVDAILFGLLVFSGILGNFLVLQSAIESPSRRLPPSDTILVHLSLANLLTSLFRTV
PIFVSDLGLDVSLAPGWCRLFMLLWVWWRAVGCWVTLALSAFHCVTLRRQHVAFGP
LAQQRERRRVWVALGLVWGVNLAFSLPALVYTTHVHGNATVELMVISCTTRPLLGC
VWEFPSAEQGSAFASTSLALNEVAPLVLMVCTNLATLHALAKHIRAVTSGGESGGNH
GELDRHVASERKAGHVIMSLVSLFVVCWALQVAAVTYYNHDGGHHAEGLLTVSHFS
ASLFVGFSPMVVALGHGKLRRRIMGMIQGWSDVVKCRHEDTEEESKSPETTGKKAK
QT

>Can_ORA4

MSEVLTVDAILFGLLVFSGILGNFLVQSAAESPSRRLTPSDTILVHLSLANLLTSLFRTVP
IFVSDLGLDVSLSPGWCRIFMLLWVWWRAVGCWVTLALSLFHCTTLRRQHMTSGPL
AQQRERRRVWIVLGLVWGANLVFSIPALVYTTHVHGNFTVELMVISCTTRPLLGCVW
EFPSSQHGSAFASSSLALNEVLPLVLMVCTNLATLHALAKHIRAVSSGAESGEAHREL
DKHLSTERKAAHVIMSLVSLFVVCWALQVAAVTYYNHDGGHHAEGLLTVAHFSASLF
VGFSPMVVALGHGKLRKRIMNMILVWSKLPKCHEEDTEEGVESPKTKGKRGKQTVFI
VHKERKVIQVKEKAQADK

>Ola_ORA4

MSKVLTLDAILFGLLVFSGLLGNTLVIYAVFQSAFETPPGRLSPSDTILVHLSLANLLTSL
FRTVPIFVSDLGLDVSLSPGWCRVFMLLWMWWRAVGCWVTLTLSIFHCTTLKRHHVS
LGPLVLQKEKRRVWIILGLVWGANLAFSIPALVYSTHVYSNATVDLMVISSTTRPLLGC
IWEFPTTQQGSAFAFTSLALNEVFPLVLMICTNVSTLHALAKHIRAVTSSMESGGSHGE
VNKLVSTERKAAHVIMLLVALFVVCWVLQVAAVTYYNHNRGLHAEGLLTVAHFSAS
TFVGFSPMVVALGHGKLRKKIMGMILVWTKAFHCSSKDTGRKRPSPKKKKLKVFDV
QEEMRVINIRECKALNDRRE

>Ssa_ORA4

MSEVLTVDAILFGFLVFSGILGNILVIHVVFQSAIESLSRRLPPSDTILVNLSLANLLTSLF
RTVPIFVSDLGLDVSLSQGWCRLFMFLWVWWRAVSCWVTLTLSAFHCATLKRQHVA
MGPLAQEHERRKVWVALGLVWGLNLAFSLPALVYTTHVQGNATVELMVISCTTRPL
LGCMWEFPSEEQGSAFASTSLALNEVVPLVLMVGTNLATLHSLAKHIRAVTSAGEAG
GGTHGELDRHVASERKASHVIMLLVMLFVVCWVLQVAAVTYYNHNRGNHAEELLT

VAHFSASVFVGFSPMVVALGHGKLRRRIMRMIAGCADRVKCQQEKIIDESKAPDKRE
RTAKQTVFTIQKEREVIK

>Sch_ORA4

MSEVLTVDAILFGLLVFSGILGNILVIHVVFQSAAESPSRRLSPSDTILVHLSLANLLISLF
RTVPIFVSDLGLDVTLSPGWCRVFMLLWVWWRAVGCWVTLALSVFHCIILRRHHVAV
GPLAQQRERQRVWIVLGLVWGANLAFSLPALVYSTHVQGNVTVELMVISCTIRPLLG
CVWEFPSIEQGSAFASTSLALNEVLPLVLMVCCNMATLHALAKHIRAVTSGGESKGTH
GELDKHVSTERKAAHVIMLLVSLFVVCWVLQVAAVMYYNHDGGQHAEGLLTVAHF
SASLFVGFSPLVVAFGHSKLRRRIMSMMLGWSKVLKCHNKDTEEGGEIPKD

>Ame_ORA4

MELLTIEAILFGFLVFSGILGNMLVIYAVFQCALDNPSHHLSPSDIILLNISMANLLTSMF
RTIPIFISDLGLKVSLDTNWCRVFMLLWVWWRAVGCWATLTLSAFHYATLKRKRVSTC
PQALRKDRRLTWGALGLVWGTNLLFSIPASVFTSHVHGNATTEVMVISCTTRPLLGC
MWNFPTREQGYAFAAASMALNEVLPLVLMVGTNLATLHTLAKHIRAVAAGPEMASG
HSNSEKKAGHVIMALVTLFVVCWVLQVAAVTYYNYERGKHTDSLLTVSQFSSSLFVG
FSPMVVALGHGKMRKKIMGMLQRWLRKALCRDAEEHKRAPEISSTINSTITQKQTHH

>Dre_ORA4

MSEVLTVDAVLFGLLVFSGIIGNIMVFDCAKLCASRHLPPSDTILVHLCLANLLTSVFRT
VPIFVSDLGLQVWLTAGWCRVFMLLWVWWRAVGCWVTLALSAFHCATLRRQHVSM
GPLGHSRERRRVWVVLAVVWAANLLFSLPALVYTTQVRGNATVELMVISCTTRPLLG
CVWEFPTFQQGYAFASSSLALNEVLPLVLMVGTNLATLQALGKHIRTVRAGGSTGAE
LDRHVSSERKAGHVIMALVALFVGCWVLQVAAVTYYNHNRGAHAEGLLTVAHFSAS
LFVGFSPLVVALGHGKLRRRISGILQSCMHRLKQTQDKPAEITEKDGRTTQSAMK

>Gmo_ORA4

MAEVLTVDAILFGLLVFTGIMGNIMVMHTVCQSAMQSQSRRMPASDTILVHLSLANL
LTSLFRTVPIFISDLGLEVTLSPGWCRVFMLLWVWWRAVGCWVTLALSAFHCSTLRR
QHVAFGPLAVQKERRRVWGALGLVWGVNLVLSLPALVYTTHVHGNATVELMVISCT
TRPLLGCIWEFPSREQGEAFASTSLVLNEVLPLVLMICTNLATLHALAKHIRAVTAAGG
DSGDMDRHLASERKAGHVIMSLVSLFVVCWVLQVAAVTYYNHDGGHHAEGLLTVA
HFAASLFVGFSPMVVALGHGKLRKRIVGKLVGCSNVVRCRGHEGEDDGGAAGAAQ
QKEKGKTVFVVQKEAASTKEKTMSKK

>Gac_ORA4

MSTRSNLHALRRSLMFSDAQRNCGKCLRLHVFQSAFESPSRRLPPSDTILVHLSLANL
LTSLFRTVPIFVSDLGLDVSLSLGWCRIFMLLWVWWRAVGCWVTLALSIFHCTVLRR

QHVACGPLAQERERRHVWIALGLVWGANLAFSIPALVYSTHVHGNATVELMVISCTT
RPLLGCVWEFPSNQQGSAFASASLALNEVLPLVLMVCTNLATLHALAKHIRAVAAGA
HPGETQKELDKHVSTERKAAHVIVSLVSLFVVCWALQVAAVTYYNHDGGDHAEGLL
TVAHFSASLFVGFSPMVVALGHGKLRRRIMNMILMWSEVLKCRKENSEERIKSPKAK
GRRGKRSVFIVQEERMVVQVKGNAQADK

>Loc_ORA4

MAQVLPVDAILFGVLVLSGIVGNVLVICAVVQSVLQNSLLRIPPSDLILANLSLANLLTS
FFRTVPIFVSDLGLEVSLAPGWCRLFMFLWVWWRAVGCWATLGLSLFHWAMLRRHS
FMSGLQAHRAELRRVCVALALVWALNFAYSLPALVYSTHSRGNTTVELMVISCTTRPL
LGCVWEFPSEVQGIAFATASLVVNELVPLVLMVGTNLASLCVLRRHIHTVAGANMEL
QGHMASERRASHVILVLVTLFVTCWGLQVTAVTHYNYNRGRQAETLLTVSHFAASVF
VGFSPLVVALGHSKLRGRLRRMLRLHCGRREGPGGEDVGDRERDREAATKTPTTTRT
CRDKS

>Lin_ORA4

MNSDKNLWGRHAEADAVAKVLTVDAVLFGLLVFSGILGNSLAIHVVFRSAIESPSWRP
PPSDTILVHLSLANLLTWLFRTVPTFVSDLGLDVSLAPGWCRLFMLLWVWWQAIMLA
LSAFYRATLKQQHVAFRPLALQWEQQWVLVALGLVWGVNLVFSLPALVYTTHIHGN
ATAELMVISCTTWPLLGCIWEFSSEEQGSVFISTPLMLNEVAPLVLMICTNLATLHALA
KNIXAVTVVEGSGGTHRELERHVASEHNVGHMIMLPVSLFVVCCVLQVAAMTXFNH
DGGHHAEGLLTVSHFSASLFVGFSPTVVALGHGKLRRXILGMILRWSDAIKCFKEESE
RENKVSNTKGQAAEEAVSVQNERSHKSRGKT

>Lin_ORA5

MDAEEWTESIIRGGMFLVGILGNNXAICSIPGPKSPIRTCEVLFISLAVCSLITNYLVDLP
DTTADFADRWFLGEFYCGMVMPTITFLPETISPQSAMAKSVRNQQHLLYLIDQCQKLV
GSLERGGVPVLLDSLRLVACLLAGRWTVTVDFSIPXFFFVTVEGVNESHEDCVDDFPH
PFVRQTYEVSTVANVIPIARTVYASVQIVITQLQNQKHIRGTSSDRILPPSLTFYFLSTSR
TNTLLPVLMRDHLVILTPAHPSTVQLTGRVPPNPAQLSQLPANSNPSSSSQVRAAKSVA
AAAAVFLVCWLTHLLLHITSNIHTSLVVXEVAGYLAASYTCIIPYMVLHGVKKLSCSC
RGQKPSVMSVLLYCFILLLIGCSVKKRIICKGWKL

>Loc_ORA5

MDTVGVIESTVRASMCFLGIMGNSMLVLHSLPSKRSHLKTSEVLFINLAASNLITNCL
VDLPDTLADIAGRWFLGEAYCGIFLFCSDLSETSSVLTTLLISVFWYQKLVGSLKRGNA
PVKLDSLGLSCGLLAASWGAALVFSVPLLSFVTVGSNRSASQDCQAHFPTHASKQTY
EATYLTLANAVPVAFMVFTNLQIVITLLTQRKRIEALKKEARLQPAAERAQPTSTGPDS
PREFSSVSTPAGNQNPSSQALADPAGKGRLDALARAAAVAPPRSRNAPRQHPGAQVR

AAMSVVAVASVFLVCWVTHLLLRIASNVNESSAIVEIASYIAASYTCIIPFIFLHGVKKL
SCRCWK

>Gac_ORA5

MDAEGWIESLIRALMFLAGILGNNWLAIRSLPGHKSSIRTNEVLFINLAVSNLITNYLV
DLPDTVADFAGHWFLGETFCAAFRFCADLSETSSIYSTFFISVFWHQKLVGSLKRGGA
PVQLDRLCLVGCLLAGSWTVAAVFSIPHVFFVAVEGRNGSKVDCVDVFPSAVARQTYE
IFYLTLANALPLAGLVFASAQIVVTLLRNKQRVQGHSSGASEEGENKSGGGRDGGVA
GTASGPGPTEDPKDPSSLTDIYTGVPASARPTGGSPGHAGTLVGDTYSGGGAPEGPGR
PSQTRAKTSSGTQVRAAKSVVAVAAVFLVCWLTHLLLRISNNIHTSSMLVEVASYIAAS
YTCIIPYIFLYGVKKLGCPCRR

>Cau_ORA5

MDAEELIESIIRALMFLAGILGNNWLAICSLPGQKSGLRTNEVLFINLAISNLITNYLVD
LPDTIADFAGRWFLGETFCGVFRFCADLSETSSIFTTLFICAYWYQKLVGSLKRGGAPV
KLDSLRLVGCLLAGSWTVAMVFSIPHFFFVKVEGVNESKEDCIDVFPSAVARQTYEIIY
LTVANALPVAGIVFASVQIVITLLQNQRRIRGHSNPDPTKEVVKEDKSLENTNDDKSVS
VVSVPGGSKDFKDSNVYTGVPASSCPKEEPHTSPLNKETDCQARAQPNPSKPGQAPP
KPSPNSGTQVRAAKSVVGVASVVVVCWVTHLLLRITSNIQTSDIVVEVASYIAASYTCI
IPYIFLHGVKKLSCSCKR

>Gmo_ORA5

MDVDDIIESAVRALMFLLGMLGNNWLAVRSIPSRLSALRTNELLFLNLAVSNLITNYL
VDLPDTMADIAGGWFLGDGYCGVFRFCADLSETSSIFSTLFISVYWYQKLVGSLKRG
GGPVQLDSLRLVGGLLAGSWGVAVVFSIPHYFFVTVEGENGSSLECNDVFPSEEAKQT
YEALYLTLANALPVAGIVYATARIVVTLMQSQKRIQGHGGNQAASEEGRAAPAAAASI
KAAGGAEGGGVAGGGEVRGAKPAPKPSPGSSNQVRAAKSVVAVASIFVVCWVTHLL
LRISSNIQTSPIVVEVASYIAASYTCIIPYIFLYGVKKLSCSCCGAKQ

>Ame_ORA5a

MNAKEWIKSTIRGFMCVSGILGNNWLGFCSLPKSRSQLRTNNILFINLAISNLITNYMV
DLPDTLELVKRWPVGRMYCSAFNFFSDLSETSSIFTTMFITVFWHQKLVGSLKHGGAP
VQMDNTRLVMALLAGSWTVSVVFSLPHLFFTSLNIQNQSSEECLEYFPSQEVKQTYE
MVFLMLANVVPIVGIVFASIQITVTLLQSQKRIKNISSRAGPRGDDQRKAASNELSSKD
YISNATSANAPNTIQKVQKSQDRSNSSSGSSQVRAAKSVVAVATVFVICWLTHLLLSIT
STIHDSIVIHEMTSYIGALYTCIIPYIYLYGVKKLTCLTCSSID

>Ame_ORA5b

MDAEGWIKSVIRGLMCVSGIIGNHWLGFSALPKSRAHLKTNDILFVNLASSNLITNYL

VDLPDMMDFTYNFLMGQMYCSVFNFCSDLSETSSIFTTLFITVFWHQKLVGSLKRGG
APVQMDNIRLVAALLAGSWIVAIAFSLPHIFLASKNNGNNTYFECLEDYPSLKAKQAY
DLMYLVFANIIPIIGIFFASIQITVTLLQNQKRINSNTTAVTTGAKNTKITPAEPSVHSNQS
SQEAVSYSNAVVPNPGQARSSSSSGSLLRAAKSVVTVATIFLICWVVHVILRLISTIQES
SLIMELASYIGAAYTCIIPYIYLHGVKKFSCTCRG

>Ssa_ORA5a

MDATEWIEAFIRGLMCLLGILGNNWLCLRSLPGPKSSLRTNEVLFINLAVSNLITNYLV
DLPDTMADFVGHWFLGEAYCCVVQFCSDLSETSSVFSTLFISVFWYQKLVGSLKRGG
APVQLDSLRLVACLLVGSWTVAVVFSVPQLFFVRMESGNESHDDCIEIFPSQTARQTYE
PLYLTFANALPIAGIAFASIQIVITLLRNQTRIQGLTSDHHKGTANSLPNNGPSVVHSSIS
SPLYQVDIGDNIVRAPADLKRSSQPPAKPSPGSGTQVRAAKSVVAVATVFVVCWVTHL
LLMMASNIHTSSLVLELASYIGSSYTCIIPYIFLYGVKKLSCSCRG

>Ssa_ORA5b

MDAKDWIEALIRGLMCLVGILGNNWLGLRSLPGPKSHLRTNELLFINLAVSNLITNYL
VDLPDTMADFAGRWFLGEAYCGVFRFCSDLSETSSIFSTLFISVFWYQKLVGSLKRGG
APVQLDSLRLVAYLLAGSWTVAGVFSVPHFFFVQVDSGNESHKDCIEVFPSQTARRTY
ETLYLTLANALPIAGIVFSSIQIVITLLRNQMRIKGLTSDHHKGTDKALPNKREKMDVS
EKYDETGIKTSDKEADLPIVASPDFPQSCRNQRISDIRPNLPGVSCSVLPCAGPSVVHSS
ISSPLYQVEVGDCRVRASPDLQRTSQPPAKLSPGSGTQVRAAKSVVAVATVFVVCWVT
HLLLRISSNIHTSSVVVEVASYIASSYTCIIPYIFLYGVKKLSCSCRR

>Dre_ORA5

MQLQDWVESSIRAFFCVTGITGNFWLALRSLPRSRSRLRPNDVLFINLAVSNLITNCM
VDLPDTLAQFLNSWLLSRNYCSVLQFSSDLSETSSIFSTMFITLYWHQKLVGSVRRGG
APVQLDNLRLVLWLLLGSWMVALTFSVPHFFIAEHDGNDTLEVCEEKFPT

>Can_ORA5

MDAEELIESIIRALMFLAGILGNNWLAIRSLPRQKSGIRTNEVLFINLAVSNLITNWLVD
LPDTMADFAGRWFLGETFCAVFRFCADLSETSSIFSTFFISVFWHQKLVGSLKRGGAP
VQLDSLCLVGCLLAGSWTVAVVFSIPHFFFVAVEGRNESEEDCVDVFPSALARQTYEIV
YLTLANALPVAGIVFASVQIVITLLKNRQRIQGHNSDATKEMKGENKSTESRNDKPVS
TVSGPEASKDPEDPASSNHIYTGVTGSSCPNGELSGHSAPLNRETNTDSGVGAPPNLP
RPSQVRAKPNPSSSTQVRAAKSVVAVAAVFLVCWLTHLLLRITNNIHTSSMLVEVASYI
AASYTCIIPYIFLYGVKKLSCSCKR

>Cge_ORA5

MDAEEWIESIIRALMFLAGILGNNWLAIRSLPGHKSPIRTNEVLFLNLAVSNLITNYLV

DLPDTMADFADRWFLGETFCGVFRFCADLSETSSIYSTFFISVFWYQKLVGSLKRGGA

PVQLDSLRLVACLLAGSWTVALVFSIPHFFFVAVEGANESHEDCVDVFPSPSAMQTYE

ALYLTLANAIPIAGIVYASVQIVITLLQNQRRIKGHNSDTKDMAKDDSKGHERSISTVS

GPGSSSDHRDTPSLSNIHISMPGSSCPVEGPSGHSSPSSPLNGEANRENRVGAPPSLSRP

SQPPAKPSPSSSTQVRAAKSVVAVASVFLVCWLTHLLLRITNNIHTSSIVVEVASYIAAS

YTCIIPYIFLHGVKKLSCSC

>Mar_ORA5

MDADDLIESTIRALMCLAGILGNSLLVIRSLPKQKSGIRTNEVLFINLAVSNLITSYLVD

LPDTMADFAGSWFLGEVYCGIFRFCADLSETSSIYTTFFICVFWHQKLVGSLKRGGAP

VQMDSLFLVFHLLAGSWTLALVFSIPHFFFVRVKGTNESHLECGEEFPNALARQTYEIF

YLTLANALPVAGIVFASVQIVITLLQNQRRIQGHKPTKEMEKDENKSNNDEKSVGAVS

DPETSKDVKDAASLTHIYTGVPASSCPNTELSGHSSHNSPIKRATKTESRVGTPPDLPKP

SQMPAKPSPSSSSQVRAAKSVVAVASVFLVCWLTHLLLRITTNILTSPIVLEVASYIAAS

YTCIIPYIFLHGVKKLSCSCKR

>Pol_ORA5

MDAEELIESIIRAFMFLAGILGNNWLAIRSLPRQKSSIRTNEVLFINLAVSNLITNYLVDL

PDTIADFAGRWFLGETFCSVFRFSADLSETSSIYTTFFISTFWYQKLVGSLKRGGAPVQ

LDSLCLVGSLLAGSWTVAVVFAIPHFFFVTVEGTNGSHEDCIDVFPNALAQQTYEIFYL

TLANALPLAGIVFASIQIVITLLQNRRRIQGHNSEATKDEKRSSETRTEERCAIADSGPG

TSKNLKDSASVNIYTGVPASSCTNGAPSGHSAPVNRETIPESKAGAPPNLPRPKQPAKP

ATSSGTQVRAAKSVVAVASVFLVCWLTHLLLRITNNIHTSSIVVEVASYIAASYTCIIPYI

FLHGVKKLSCSCKR

>Xma_ORA5

MDADKLIQSIVRILMFIAGILGNNLLVIASLPRKKSEIRTNEILLINLAVSNLITNYLVDIP

VTMEDFAGRWFLGLSFCGIFHFSSDLSETSSLFTTLIICIFWHQKLVGSLKRGGAPVQL

DNLHLLGCLLAGSWTLSAIFSMPRLFFVSQEVKNESHENCIDVFPDVLSRETYEVIFLS

LANALPVAGIWVASIQIVLTLLQHRKRIQSVSSHRVKVKQDKSERSVSSVSVQDSHKD

LKESSSPTEHVSSCPNGKCLGKSPRSSPLANKTDSRAEAQPNHSVCQSSQIPSKQSNNA

SSQVRAAKSVVAVATVVLLCWLTHLILRISNSMQDSPLMMELASYIGAAYTCIIPYIFL

HGVKKLSRLCK

>Tni_ORA5

MDTKELAESIIRGMMFLAGILGNNYLAARSFPTQRTSIRTNEVLFINLAVSNLITNYLV

DLPDTMADFAGRWFLGETYCGIFRFCADLSETSSIFTTLFISVFWHQKLVGSLRRGGAP

VQMDNLCLVACLLAGSWTVAAVFSVPHFFFVKVEATNESSEDCIDVFPNKLAKQTYEI

IYLTLANIFPVAGIVFASMQIVVTLLQNQRRIQSHSSNPTQTTNRTEDRSSCTSGKDSTL

TSPVYTGVPPVSADGGALAQSSGEPQPGGTARSHQECPRPGQAPTKPTLASGSQVRA
AKSVVAVASVFLVCWLTHLLLRISNNVHTSSVVVEVASYIAASYTCIIPYIFLHGVKKL
YCSCKR

>Tru_ORA5

MDAEELVESIVRGLMFLAGVLGNNWLAVRSFPTQRSSVRTNEVLFLNLALSNLITNYL
VDLPDTVADFAGRWFLGETYCGIFRFCADLSETSSIFTTLFISVFWHQKLVGSLKRGGS
PVQMDSLCLVACLLAGSWTVAVVFSIPHFFFVVEGSNESSEDCIDVFPNKAAKQTYEII
YLTLANAVPVAGIVFASVQIVITLLRNHRRIRSHGPDPTKISNEPKDRSSDPGPTPPSQV
YTGVPPSGGAVAQSSREPQPGGTDRTPEGGPRPSQAPAKPTMASSSQVRAAKSVVGV
ASVFLVCWLTHLLLRITNSVHTSSLVVEVASYIAASYTCIIPYIFLHGVKKLHCSCKR

>Oni_ORA5

MEELIESIIRGLMFIAGILGNNWLAICSLPRHKSSIRTNEVLFINLAISNLITNYLVDLPDT
MADFAGRWFLGETYCGVFCFCAGLSETSSIFTTFFISVFWHQKLVGSLKRGGAPVQM
DSLCLVGCLLAGSWTVAVVFSVPHFFFFTLEGANDSHEDCIEIFPNPNARQTYEAIYLT
LANALPMAGIVFASAQIVITLLQNHQRIRSHNSDQTKEMVKEERKRFESKRNKASVSII
SGPTSSKDLRDSTSTNHIYTGVPAPSSPNRQPSGHSLRNAPQNCSVGAQPNLSRPSQIPS
KPHPNSSTQVRAAKSVVAVASVVLVCWLTHLLLHITNNIHTSSIVVEVSSYIAASYTCII
PYIFLHGVKKLTCSSKR

>Ola_ORA5

MELNKPIASVISALMFLASILGNNWLAVASLPKDRSAIRTNEVLFINLAVSNLITNYVV
NLPETMADIADNWFLGETFCCVFLFSIDFSETSSLFSTFLISAFWHQKLVGSLKRGGAP
VQLDNLCLVGFLLAGSWTVCAVFSIHHFFLASVEGINGSHRYCVDAFPSALAEQTFDII
FLTVANVFPLVGIIVASFQIVVTLLQSQKRIGGHTSVSPKEMIREDKSSQSKKQVMGPL
KGLKVVRTYKSEPVTSSVTLPQTSSQNKTTGSNCSLGAPANHSKPSKANPNSSTQVRA
AKSVVAVGSVVLVCWLTHLLLHITNTVHSSQSTLEVAGYITASYSCIIPYILLHGVKKLT
CSHKR

>Ola_ORA6

MEGIYVNLLGLRIAVSFTGLVGNVCLILSIIHVKWSHIKSFEVFLLGLAAANLEEIVILN
VYDAFMLQTSSSDTWWCRFLKFMTMFGETASIFFTVIISIFRYQKLGVSLSVHPDRIGV
AQLLSGVCVMFSFLLSFPVVAIKPAALTESAANNSGGGCPADSFHCGKNYCPAPNRAY
KYLFILVSYLLPLIVITVTNCLILAVLLVQRRTITPEISVHQSNHSHTNGRDLRFQHSMM
AVVAAMGLFLVNWTFYLLFLFLLKPNNLPSWREIEFFTLTSYSCFSPYVYGIGHNLFSL
ENFKIIRNKF

>Oni_ORA6

MAELSVNLLGLRLVFSSVGLMGNTILIASIIKINFFHIKSFEIFLFGLAAANLWEIVITNIY
DIIILQTSSTATGTWSCYLLEFMTVIGEINSIFFTVLICIFRYQKLRDVNTRVNFPLFLDNI
RSAWMVSGISVMLSVLLSVPMFVIDQESKAENVTRNSSVCPPDFFHCTQNHCPVFNSI
YKYLFIVLCHLLPLIIVTVTSCLILAVLLSQRKTVTPAVNETGSSQFSRKSKDTKIQWSTI
AVLGAMGLFQVDWTIYLIFQLAFNPYEFLFWSEVQFFITISYTSISPYMYMIGHNMIPL
HSCKKGSFKGTVSDFVEVI

>Tru_ORA6

MLGLSVQLLATRIIISCIGIFGNVFLIVSVVQNKFSQIKSFELFLLELAAANLEEILIVNIY
DMIILQTSFATVGTWSCRLLKFLTMLGENASILVTVLISIFRYQKLRDASRRVNLPIYLD
SIRSVWTVSGILTVFTILLSSPIFVLNIKETSQNFTNNGSGCPPDFFQCNKEDCPELNGIY
KYLFILLFNLLPLIIVTVTSCLIIAVLLSQRKTVTPVESGSSQISRKSKGLKFQRSTIAVLT
AMGLFQVDWTVYLIFQLTISPGDSSSWAEIKFFISTSYTSISPYVYGIGNNLFSLKKLRK
N

>Tni_ORA6

MLALSVQLLATRIIISCIGIFGNVFLIISVVQTKFSRIKSFELFLLELAAANLEEIFIVNVY
DIILLCTSYATVGTWSCRTLKFLTSLGETASILITVLISIFRYQKLRDASRRVPIYLDSIRS
AWTVSGILLMFTVLLASPIFVLNIKEMSQNVTINGSGCPPDFFQCNKDNCPELNGIYK
YLFILLFNLLPLIIVTVTSCLIIMVLLSQRKTVAPVVNASSQTAQRSKCQKFQRSTIAVLT
AMGLFQVDWTLNLIFQLTSSPGAFTSGAEIKFFISSSYTAISPYVYGIGNNLFSLKKFRK
T

>Xma_ORA6

MSHLSVDLLGLRLFVSCVGLVGNIFLILFIFQTRVSHIKSFELFLLGLASFNLEEIVSINV
YHVIILDTVFTTTGAWWCRLLKFMTTFGEIASILFTVVICIFRYQKLRDVDHRGSLPICL
DSIASAWTMSGVCVTLSALLSLPMFAIAFRGSVENVTENREGCPTDFFQCGENYCPIL
NCVYKYLIMLLCHLLPLIIVTVTSCLTIVVLLGRTNTVTPANDIISPDHHPGKSHGFYRS
TVAVVAAMGLFQVDWTLYLILQWTFSPSDCPIWVEIEFFISASYMSISPYVYGIGGHLF
SLENCKHLLKR

>Ssa_ORA6

MVDTLELLVFRIFISTVGIVGYVVLILSLIKNQISCLKTFEVFLLGLAASNLVAPNHNVH
PHHLVLPAPQVLHNGLAFLGQWWAISRLLLVLLVSYTAEGYMSLRPGQAEIKLCVWL
YLFGMLVFGKIASILFTVLFSIFRYQKR

>Pol_ORA6

MNGISVSFLSLRILISCIGLVGNVILILSIIQTKFFRVKSFEFFLLGLATANLEEIVIMNIYD
TVILEGYFIITSTWPCRILKFLTVFGEISSILFTVLISIYRYQKLRDASKRASLPICLDSIRS

AWMVSGVCVALSLLLSFPSFVIKLHGPAHNATGDRGSCPPDFFQCDKHFCPLINCIYK
YMFLMMCNLLPLIIVTGTGCLIIRVLLSQGKRVTSVASVSMSSQNTKKSKGPRLQRSLI
AVLAAMGLFQLDWTLYLIFQLAFMPTDLPFWDEMEFFISASYASISPFVYGIGNNLFSV
KNLMRKWG

>Mar_ORA6

MDGYFVDLLVLRIIISCIGFVGNVLLIFSVIIHSKLSRVKSFELFLLGLATANLEEILIMNI
SEIIVVKLSFFQNGIWICRSLRFLTVFGEITSIFFSVLISIFRYEKLRDTNKRANFPVFLDSI
RSAWMVSGVCVMISTLLSFPIFVLDVQNPAVNVTINSSTCPPDFFQCSKKDCPTPNLFY
KYLFIVMCNLLPLIIVTVTSCLIIMVLLSQSHTVTPEEIVHGLSHHGNKKLQHSIIAVLA
AMGLFQINWTFYLIFQLIFNATDFPFWAEIERFITTSYISISPYVYGIGSHLFSVKNLIKS

>Cge_ORA6

MVSFSPELLGLRIFISVIGIMGNVFLILSIIQTKFSRVKSFELFLLGLAVSNLEEILIVDIYD
VVMRQASSGSRNWTCRSLKFLTALGETTSILFTVLISVFRYQKLRDAHKRVNLPIFLDS
LRSAWMVSGICVSLAILLSSPIYVLNLDEHTDNNTTSNGIGCPPDFFLCHKDNCPTVNR
LYKYMFLLVCNLLPLVIITVTSCLILTVLLSQRSMITPVLSVSGSGQPGKKSKGPRLQRS
TVAVLAAMGLFQVDWTLYLVFHLAFHPSDFHFWSEMEFFISTTYTSSSPYVYGIGNNL
FSLKNIIK

>Can_ORA6

MAAAAVNLLGLRVFIVCVGLVGNVVLILAIIQTKFSRVKSFELFLLGLASANLEEIVIM
TVFDVVVLQTSSGVASWSCRSLKFLTKFGEVASILFTVLISVFRQQKLRDADKRVNQPI
YLDGVRSAWMASGACVLLAALLTLPVFVLGLEGAAGNATGSGGGGGGCLPDFFECS
EDRCPAVNAAYKYLFLVLCNVLPLAVVTATGCLIVAVLLGRRSKVTPAPASGGRRGGG
SSFHRSTVAVLAATGLFQLDWTLYLILQLTVIPTDRDFWAEAEILISFSYMSLSPYVYG
MGNNLFSLRSFRRK

>Ame_ORA6

MESFIFGLLVLRIMLSVIGVLGNTVLIVSILQMTRLKTFEVFLLGLAVSNLEEIMIVDIY
DMIVLRSTHSISILSCGVLKFMTLSGEVASIFFTVLISIYRYQKLHNAAMRIITPIFMDSM
KIGVGLSLLCVLVAVLASVPTYIINLDSWHHMYNSTITDCPADFFQCPRDNCPILNNIY
RFLFIFFCYLIPLVIVTGTSSLIIRILMIQQKVAELHHNSEPATIAANNDHHHHHHNHHH
HHDNHHHHHDHHHHDHTNVFHRSTIGILAAMMIFQVYCILYLARHLAFNLYDFPAWS
ELEFFIATFYTALIPYVYGMGHNFFSLKHFRRQ

>Sch_ORA6

MVGWSVNLLGLRVFVSCIGLVGNLFLILSIIQTNFSQIKSFEVFLLGLAAANLVDIVIVN
IYDIIIFQTSSTTTTGSWLCHSLKFLTVFGEITSILFTVLISIFRYLKLRDTDKRVNLPICLD

SVRSAWTVSGVCVMLSTLVSLPIFVINLQDPAENVTRSSSGCPPDFFQCSKNDCPILNR
FYKYLFILVCNLLPLIIVTVTGCLIITVLLSQMKTVTPAVSGSSQFGRKSKSLRRQRSTIA
VLAAMVLFQVDWTLYLIFQLTFSPTDFPFWAEMQFFVSTSYTSISPYVYGIGNNLFSLN
NFIKK

>Dre_ORA6

MVMEQIQVNLLSLRLFISIIGVVGNTLLLVSILHTHTHLKSFELFLLALCSANLQQLVM
VDVYDVLLLCSPSCIGVCSCRALRFLTVFGEVCSVLFTALISIYRHQKLHDVFSHVNVP
VLLDSLRWAVCMCVLCVCVALAFGLPTLLVNTHWSVSNSSLERCPVDFFQCPSSSPCL
THIYKYVFLLVCVVLPLLVVTVTSV

>Cau_ORA6

MAAFSADLLGVRVTISCIGLVGNVFLIISILQNRLSRVKSFELFLLGLAAANLEEIVIINI
YDAIILQTSSTVISSWSCRLLKFMTVFGEITSILFTVLISIFRYQKLRDVNKRANLPIYLD
NLGSACLMSGVCVVLSTLFSLPIFVMNLQDSAGNITGITSRCPPDFFLCSIDNCPLLNR
LYKYVFMLVCHLLPLIIVTVTGCLIITVLLGHKKTVIPIDSVSGSRQLGRTNKDTRFQRS
TVAVLAAMGLFQVDWSFYLIFQLTLSQTRVPFWAEIEFFISTSYTSISPYVYGVGNNLFS
FKNFLKK

>Gmo_ORA6

MAKVEYKTMITLVTIRFVMSLIGIMGNMFLVFVIFQTKISRIKSFEVFLLGLAVSNLEEL
VVVDFYEVIMLIGHIQNSLLCRTMKFLNLLGEVSSILFTVLICVFRYQKLRDAEKRGN
APIFLDSRKSAWVVSGLCMLLSVMLGLPVYFVRIDTHVEADNGTSCSPDFFQCHEHF
CPPLNRFYKYLFLVSCNLLPLLAVTVSSSLIVKVLLGQKRVVAPALGASGPPGKKSKGP
RLQRSTVGILTAMGVFQIDWTMYLVFHLAFSPVNVPLWGDIEFFITTSYTTLSPYVYGI
GYDLFSLRYFIKR

>Gac_ORA6

MVGLSVDLLGLKVFILCVGLMGNVFLMVAVAQTKFPRVKSFELFLLGLAAANLEEIAI
TTVFDVDFLQASSRGVDTWSCRSLKFLSKFGEVASIFFTVLISVFRQQKLSDAAKRAN
LPIYLDSIGSARMASGVCVLLATLLSLPVFAIEPKEPAGNATGNATGNATGNAIGCPPDF
FQCSKSRCPALNGLYKHVFILVCNLLPLAVVTVTGCLILAVLLGQRSTVTPASVGSRSG
GSTLRRSSVAVLAAMGLFQVEWTLYLILQLTAVYVDFAFRAEAELLISFSYTCISPYVY
GIGNDLFSLKNFKRN

>Loc_ORA6

MDLMNPVLLVLRYLISIVGIIGNITLVVSILSHSHMKTFEIFLLGLSFSNLEGIFLVSIFDIT
TRLALQSLEEWSFKILRFMASLGETATIFFTVLISVFRYQKLRHAEARGNLPTSWDNTS
TAWALSGMSLFLSFCFCLPGYFIESDERVDNHTRSRNFLTDPFQCPRINCPAINLIYKTL

FLLFSNLIPLLIITATSGLILKVLLHRRKTVSDVYDSSHHHHQNLYFSKSTKTVLAAMCI
FQLDWIMYLVLHLAFDSSKMDNWSEIEFFIVTTYTTISPYVYGIGTNIFSCRQIVKVLS
CLSFSACGLYRQHSMCAKWNQ

>Loc_ORA7

MDLQNLAKAVATLLQNMVGIPANLTVLGVFVHVARTERRLLPTDAIVSHLVSVNLLLI
LTRGIPQSLSALDYRGFYDSATCKFLIFTYRTTRAMSISLTFVLSAYQCITIAPASSRLSR
LKPWLYRCLLPLNLFFWLLNGGTTYTSILYTSQVRNTTLSTNTLNLGYCLVVFPSEES
YFANGVMYLTRDLFFVILMVLASFYILLLLYRHQRRVKGLQSSNMSQGSRAETRAAK
TVVTLVTLYVLFFGIDNLIWAYTLTTEKVPLLMNDVRVFFSSLYASVCPVVVIVSNRKV
NRRLSCIKLA

>Loc_ORA8a

GLNVAVCQASLLYSSAPTNSSLSEYTLNLEFCIVAFPSFEAYMGNGVMHIVRDFVFVG
MMASAGGYIVVILYRHRKQTRGLQGAARMQRKTVEASKAVLTLIAMYVILFGLDNV
VWIYTLCVSRVHPIASDTRVFFASCYSALSPIFIITTNKKIVASLSCCKGKDQKHLIAEST
VSHLSPGQ

>Loc_ORA8b

VMYIVRDFVFVGMMASAGGYIVVILYRHRRQTRGLQGADRMQRKTVEASKAVLTLI
AMYVILFSLDNVMWIYPLCVSHVHPIVSDIRVFFASCYSALSPVFIITTNKKIAASLSCC
KGKDQKHLIAESTVSHLSPGQ

Supplementary Figure S1

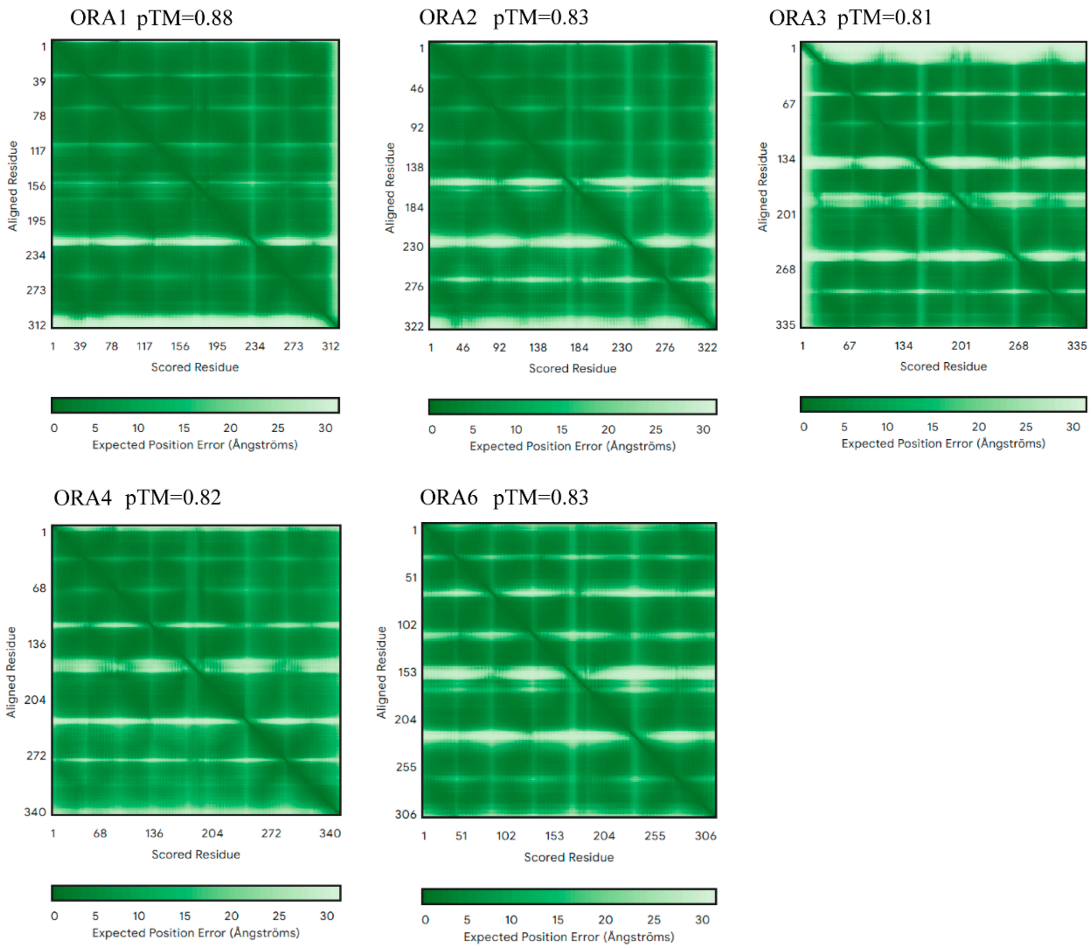

Figure S1. Predicted template modeling (pTM) score of mandarin fish ORA proteins. The pTM scores obtained for the 3D structures of the mandarin fish ORA proteins using AlphaFold 3, which measure the overall accuracy of the structures, are all greater than 0.8.

Supplementary Figure S2

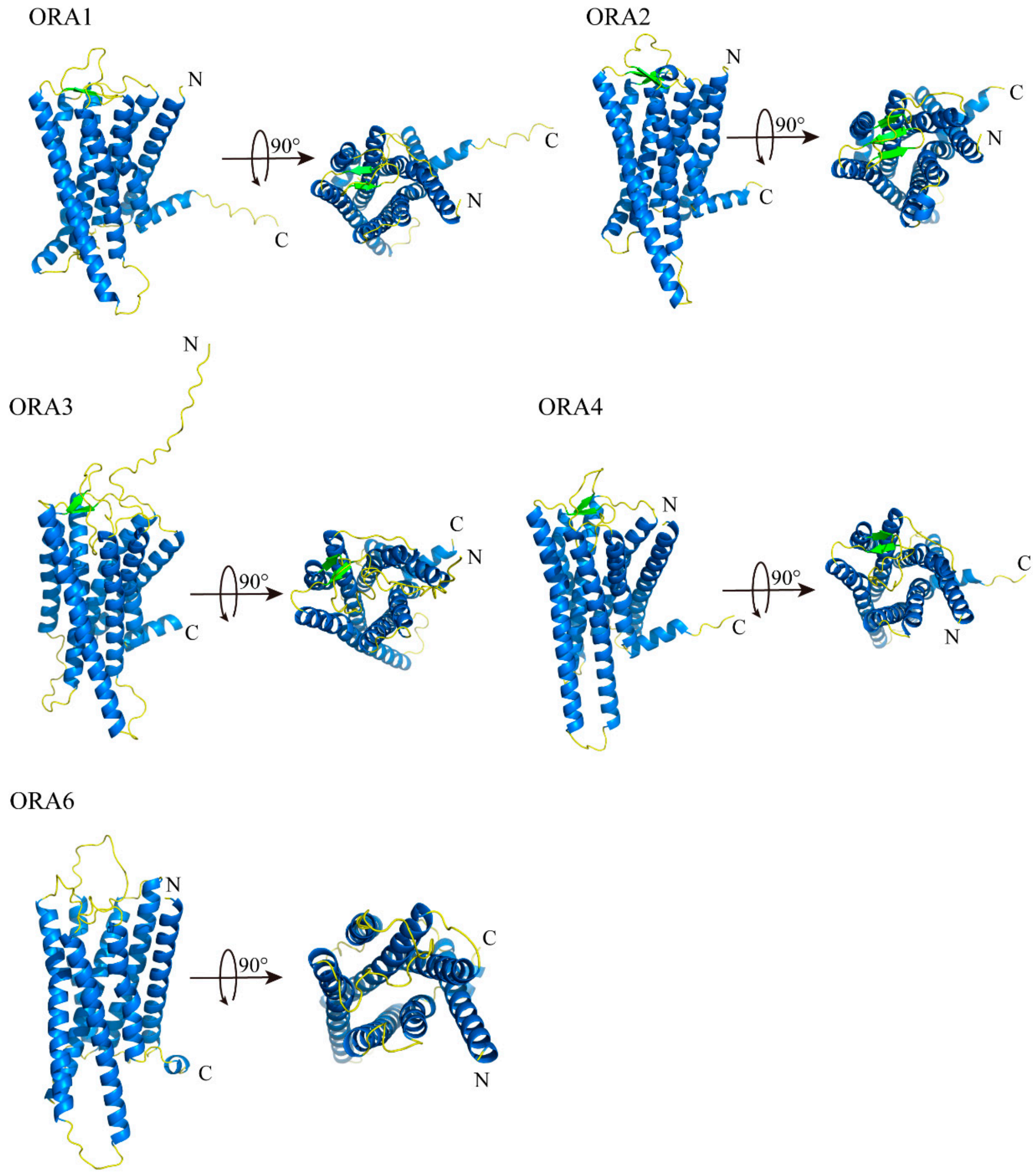

Figure S2. 3D structures of ORAs predicted by AlphaFold 3.
